# Identification of Arginine-Vasopressin Receptor 1a (Avpr1a/*Avpr1a*) as a Novel Candidate Gene for Chronic Visceral Pain Sheds Light on the Potential Role of Enteric Neurons in the Development of Visceral Hypersensitivity

**DOI:** 10.1016/j.jpain.2024.104572

**Published:** 2024-05-18

**Authors:** Leena Kader, Adam B. Willits, Sebastian Meriano, Julie A. Christianson, Jun-Ho La, Bin Feng, Brittany Knight, Gulum Kosova, Jennifer J. Deberry, Matthew D. Coates, Jeffrey S. Hyams, Kyle M. Baumbauer, Erin E. Young

**Affiliations:** * Department of Anesthesiology, Pain, and Perioperative Medicine, KU Medical Center, Kansas City, Kansas,; † Neuroscience Graduate Program, KU Medical Center, Kansas City, Kansas,; ‡ Department of Cell Biology and Physiology, KU Medical Center, Kansas City, Kansas,; § Department of Neurobiology, University of University of Texas Medical Branch, Galveston, Texas,; ¶ Biomedical Engineering Department, University of Connecticut, Storrs, Connecticut,; ∥ Department of Neuroscience, University of Connecticut Health Center, Farmington, Connecticut,; ** Division of Statistical Genetics,TenSixteen Bio, Suffolk, Massachusetts,; †† Department of Anesthesiology & Perioperative Medicine, University of Alabama at Birmingham, Birmingham, Alabama,; ‡‡ Department of Medicine, Division of Gastroenterology & Hepatology, Penn State College of Medicine, Hershey, Pennsylvania,; §§ Department of Gastroenterology, Connecticut Children’s Medical Center, Hartford, Connecticut

**Keywords:** Irritable bowel syndrome, disorders of gut-brain interaction, genetics, visceral hypersensitivity, enteric nervous system

## Abstract

Chronic abdominal pain in the absence of ongoing disease is the hallmark of disorders of gut-brain interaction (DGBIs), including irritable bowel syndrome (IBS). While the etiology of DGBIs remains poorly understood, there is evidence that both genetic and environmental factors play a role. In this study, we report the identification and validation of arginine-vasopressin receptor 1A (*Avpr1a*) as a novel candidate gene for visceral hypersensitivity (VH), a primary peripheral mechanism underlying abdominal pain in DGBI/IBS. Comparing 2 C57BL/6 (BL/6) substrains (C57BL/6NTac and C57BL/6J) revealed differential susceptibility to the development of chronic VH following intrarectal zymosan instillation, a validated preclinical model for postinflammatory IBS. Using whole-genome sequencing, we identified a single-nucleotide polymorphism differentiating the 2 strains in the 5′ intergenic region upstream of *Avpr1a*, encoding the protein *Avpr1a*. We used behavioral, histological, and molecular approaches to identify distal colon-specific gene expression and neuronal hyperresponsiveness covarying with *Avpr1a* genotype and VH susceptibility. While the 2 BL/6 substrains did not differ across other gastrointestinal phenotypes (eg, fecal water retention), VH-susceptible BL/6NTac mice had higher colonic *Avpr1a* mRNA and protein expression. These results parallel findings that patients’ colonic *Avpr1a* mRNA expression corresponded to higher pain ratings. Moreover, neurons of the enteric nervous system were hyperresponsive to the *Avpr1a* agonist arginine-vasopressin, suggesting a role for enteric neurons in the pathology underlying VH. Taken together, these findings implicate differential regulation of *Avpr1a* as a novel mechanism of VH susceptibility as well as a potential therapeutic target specific to VH.

Irritable bowel syndrome (IBS) is one of the most common disorders of gut-brain interaction (DGBIs). Patients report persistent abdominal pain in the absence of structural abnormalities or ongoing inflammation,^1–4^ along with alterations in bowel habits that can include diarrhea, constipation, or a combination of the 2. Prior work suggests that only 30% of people with DGBIs will seek medical care, but patients with unexplained abdominal pain are 40% more likely to receive an opioid prescription than patients with structural gastrointestinal (GI) disorders (eg, inflammatory bowel disease).^5^ Notably, approximately 60% of patients with an opioid use disorder report the presence of at least 1 chronic pain condition prior to their first opioid use disorder diagnosis.^6^ This reflects both the lack of visceral pain-specific therapeutic options and the current knowledge gap surrounding the underlying mechanisms of DGBI-related visceral pain.

Visceral hypersensitivity (VH) refers to increased sensitivity to mechanical stimulation of the visceral organs (eg, bowel) and is a driver of chronic abdominal pain in DGBIs. VH of the distal colon/rectum occurs in up to 90% of patients with IBS,^7,8^ so a valid preclinical model should manifest VH in the absence of ongoing inflammation and structural disease of the bowel.^9^ In contrast to other preclinical chemical models of VH,^10–18^ intracolonic instillation of zymosan (ZYM), a protein-carbohydrate complex found in the cell walls of the yeast *Saccharomyces cerevisiae*, results in VH in the absence of structural damage or ongoing inflammation, paralleling the clinical presentation of IBS.^4^ ZYM-induced VH has been studied preclinically using both rat and mouse models,^11,13^ yet differential susceptibility among substrains has yet to be fully leveraged to explore genetic determinants of VH susceptibility. To identify the contribution of novel genes/genetic variants involved in the susceptibility of VH, we compare the responses of 2 murine substrains that differ genetically due to the elimination of large portions of the genome; otherwise, biologically, the 2 strains are similar.^19–23^

Genetic studies evaluating IBS have focused primarily on predictors of the diagnosis or associations between polymorphisms and IBS-subtype classifications but have not explicitly evaluated the genetic underpinnings of abdominal pain persistence and/or severity. Given that abdominal pain represents the key symptom across all IBS subtypes,^24^ understanding the genetic contribution to VH may provide insight into novel therapeutic targets for treating abdominal pain in this population. Capitalizing on differential susceptibility to ZYM-induced VH between 2 commonly used C57BL/6 (BL/6) substrains (BL/6J from Jackson, BL/6NTac from Taconic) and whole-genome sequencing,^21^ we identified arginine-vasopressin receptor 1A (*Avpr1a*) as a high-priority candidate gene for VH. In this study, we use the ZYM-VH model using molecular, physiological, and calcium (Ca^2+^) imaging approaches to identify whether differential expression of *Avpr1a* corresponds to alterations in specific neuronal responses between strains, supporting a role for *Avpr1a*/*Avpr1a* in VH susceptibility. We additionally measure the relationship between visceral pain intensities experienced and *Avpr1a* expression in colorectal biopsies from patients. Our findings support *Avpr1a* as a novel visceral pain-specific VH therapeutic target and a potential VH susceptibility biomarker in DGBIs.

## Methods

### Subjects

#### Mice

Adult (8–10-week-old) male C57BL/6 mice from Jackson Labs (Bar Harbor, ME) (C5BL/6J; BL/6J) and Taconic Biosciences (Germantown, NY) (C57BL/6NTac; BL/6NTac) were housed at the University of Pittsburgh and University of Kansas Medical Center in animal facilities on a 12:12 light:dark cycle with food and water available ad libitum. Animal use protocols conformed to National Institutes of Health guidelines and were approved by the Institutional Animal Care and Use Committees at the University of Pittsburgh and the University of Kansas Medical Center and conformed to the Committee for Research and Ethical Issues of international association for the study of pain.

#### Human Subjects

Outpatients presenting for an elective colonoscopy at the University of Pittsburgh Medical Center were approached about participation. To be included in this study, participants had to be between 18 and 70 years of age, able to understand, speak, and write English, and make their own health care decisions. Written informed consent that was Health Insurance Portability and Accessibility Act-compliant, and approved by the University of Pittsburgh IRB (protocol no 11070169), was obtained for all participants.

### ZYM Model of VH

**Figure 1.**
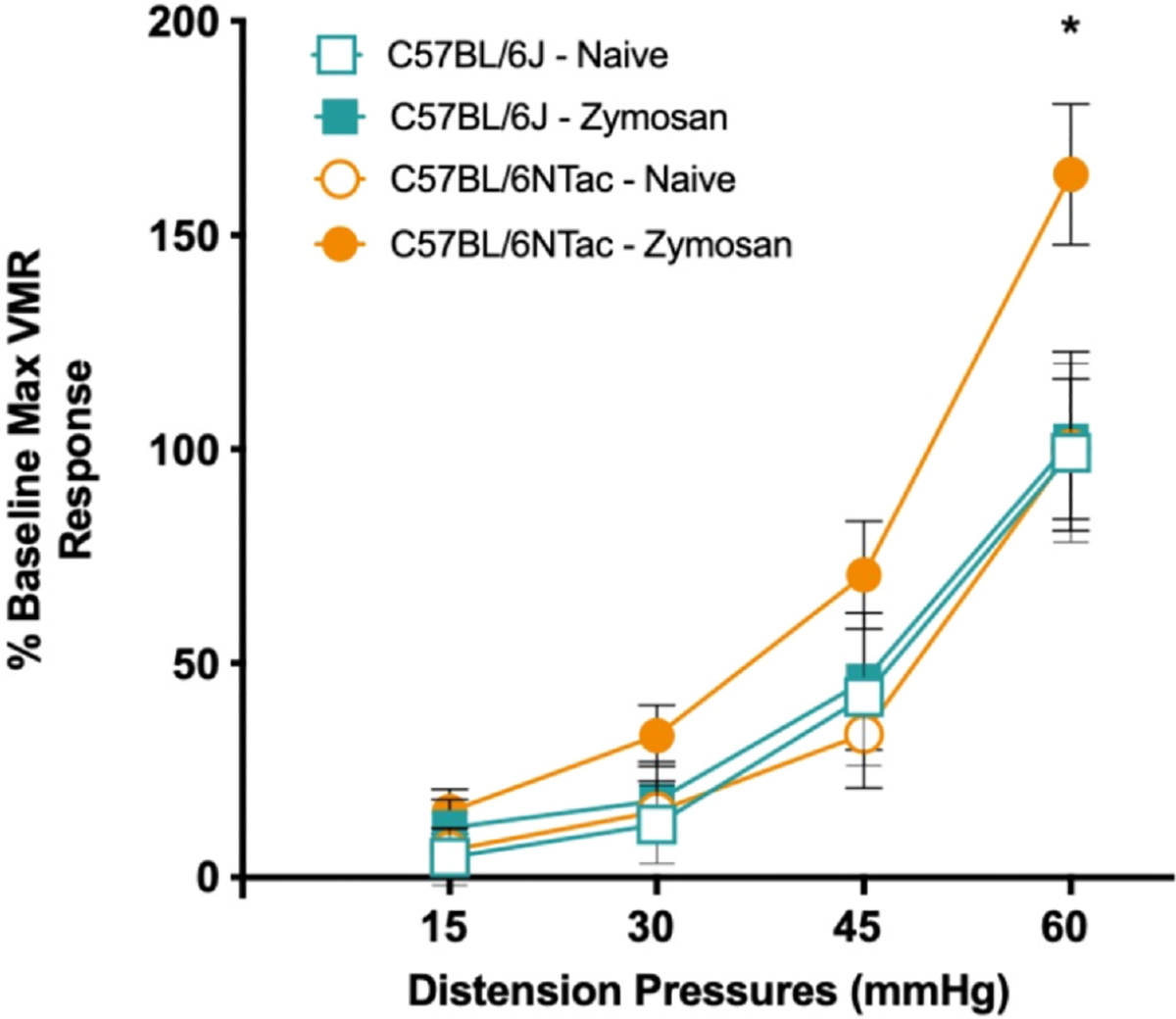
C57BL/6NTac, but not C57BL/6J, mice develop ZYM-induced VH. Visceromotor (EMG) responses were measured during colorectal balloon distension pre- and post intracolonic ZYM instillation for each strain. The graph shows the following groups: BL/6J-naïve (before ZYM, open teal square), BL/6J-ZYM (21 days after ZYM, real square), BL/6NTac-naïve (before ZYM, open orange circle), and BL/6NTac (21 days after ZYM, orange circle) mice (n = 7–10). Each mouse was normalized to its own naïve/baseline response to the maximum distension pressure of 60 mmHg. ANOVA on the cumulative AUC across all distension pressures revealed a significant main effect of strain (F [1, 11], *P* = .015) and a significant Strain × Time interaction (F [1, 11] = 6.863, *P* = .024). Follow-up 1-way ANOVA considering only VMR to 60-mmHg distention pressure confirmed a significant main effect of group (F [3, 38]) = 3.493, *P* < .05). Post hoc group comparisons using Fisher’s LSD also confirmed that the VMR to 60-mmHg distention pressure differed between B/6NTac-ZYM and all other conditions (all *P* < .05). * Indicates significant difference between C57BL/6NTac-ZYM and all other conditions. Abbreviations: LSD, Least significant difference.

Intracolonic treatment with saline (SAL) or ZYM was performed daily for 3 consecutive days as described previously.^25^ Mice were anesthetized with isoflurane gas anesthesia (2% for induction followed by .50–1.0% for maintenance) followed by a transanal administration of .1 mL of SAL or ZYM (CAS #: 58856-93-2, Sigma-Aldrich, St. Louis, MO) solution, at 30 mg/mL, via a 22-gauge gavage needle. Mice were inverted slightly for 60 seconds following installation to prevent leakage following administration and then returned to the home cage for recovery from anesthesia. Note that for Fig 1, ZYM-treated mice were compared with their own naïve (no SAL) baseline scores prior to treatment with ZYM to account for any variability between the strains at baseline and to compare to prior work using repeated measures. All other figures compare SAL- versus ZYM-treated mice. We have subsequently compared data from naïve mice and SAL-treated mice and found no significant differences in visceromotor response (VMR) at any of the distension pressure in the 15 to 60-mmHg range. The exception to this is that repeated measures of the noninvasive measures of body weight and fecal water weight were conducted for groups across the time period during which VH was developing (21 days post ZYM).

### VMR to Colorectal Distension

Evaluation of sensitivity to colorectal distension (CRD) was conducted at baseline (prior to ZYM instillation) and again 21 days after the final ZYM installation based on prior work identifying this as a time when VH is established.^11,26,27^ Mice (n = 7–10/condition) were anesthetized with isoflurane gas and the lower abdomen was shaved and washed with betadine, followed by 70% ethanol. A vertical skin incision, approximately 2 cm in length, was made in the lower abdomen to expose the abdominal musculature, and the skin was separated from the underlying muscle around the side of the abdomen. A pair of stainless-steel wire electrodes were implanted in the right abdominal musculature and then secured with sutures (7–0 Prolene) at the site of contact with the muscle. Electrode wires were then secured in place by a common suture anchor to the muscle wall of the lower abdomen. The free ends of the electrode wires were tunneled subcutaneously along the side of the abdomen internally and externalized at the nape of the neck, where the ends were anchored with internal sutures for access during testing. Both the abdominal and neck incisions were sutured (4–0 Vicryl), the wounds cleaned, and subjects were administered a subcutaneous injection of 2 mg/kg of buprenorphine in sterile saline. Buprenorphine was administered every 12 hours for the first 48 hours post surgery (a total of 4 administrations), and mice were monitored for signs of distress. Mice were placed in the home cage on a heating pad set to low and monitored for 1 hour post surgery to ensure recovery. One week after electrode implantation, mice were transferred to a separate room for VMR testing. Under light anesthesia (inhaled isoflurane, 2%), a custom-made, catheterized polyethylene balloon (1.5-cm long, ~.9 cm) was inserted through the anus until the proximal end of the balloon was .5 cm from the anal verge (total balloon insertion, 2 cm) and secured to the base of the tail with tape. The mouse was then placed into a plastic cylinder to limit movements, and the free ends of the electrode wires were attached to a differential amplifier (Model 1700, A-M Systems, Sequim, WA). Mice were allowed to recover from anesthesia for 30 minutes. Then electromyographic (EMG) activity, the VMR, was recorded using Spike2 software (Cambridge Electronic Design, Cambridge, UK) for 10 seconds before (resting) and during 10 seconds of phasic CRD (15, 30, 45, or 60 mmHg). Mice underwent 3 trials at each pressure with a 4-minute intertrial interval. Responses to distension were quantified as the total area under the curve (AUC) of EMG activity during balloon inflation minus resting activity. The 3 responses to a given distension pressure were averaged for use in all statistical analyses as previously reported.^11^ All scores for each mouse were normalized to their maximum response magnitude at baseline, reflecting our focus on differential sensitivity to VH and controlling for baseline differences in VMR to CRD.

### Genomic Comparison of Pain- and Nociception-Related Genes Between BL/6J and BL/6NTac

A systematic mini-review of all genes where variation had previously been associated with pain, hypersensitivity, and/or nociception was performed. This included individual candidate genes and genes from genomic loci identified in the mouse (eg, quantitative trait loci [QTL]). This gene list was then compared to a list of all differential single-nucleotide variants (SNVs) between the BL/6NTac and BL/6J single-nucleotide polymorphisms (SNPs). This list was produced from publicly available data included in a recently published work by Mortazavi and colleagues.^21^ Data and code availability of whole-genome sequencing and differential SNP data can be found under the “Key resources table” in the deposited data section.^21^ Differential SNPs were then identified to either be within a known gene sequence (ie, 5′ flanking region, exon, introns, or 3′ untranslated region) or near a known gene (defined as 18 kb upstream and downstream of the coding sequence) using Ensembl Archive Gene Browser (Nov-2020; Genome assembly GRCm38.p6 [GCA_000001635.8]). We then generated a finalized candidate gene list where variation(s) within pain/nociception genes differentiated the 2 strains.

### Histology and Immunohistochemistry

Mice (n = 5/condition) were overdosed with inhaled isoflurane (> 5%) and perfused transcardially with ice-cold Hanks Balanced Salt Solution (HBSS) on day 21 post SAL or ZYM treatment. Approximately 4.5 cm of the distal colon was collected and post-fixed in 10% neutralized formalin fixative. After fixation, the distal colon tissue was embedded in paraffin wax, cut into 8-μm-thick cross sections using a microtome, and mounted on glass slides. Sections were deparaffinized, rehydrated, and washed briefly in distilled water. For hematoxylin and eosin staining, slides were stained in Harris hematoxylin solution for 8 minutes and subsequently washed in running tap water for 5 minutes. Samples were differentiated in 1% acid alcohol for 30 seconds and washed in running tap water for 1 minute. Samples were then placed in .2% ammonia water or saturated lithium carbonate solution for 30 seconds to 1 minute and then washed in running tap water for 5 minutes. Afterward, slides were rinsed in 95% alcohol for approximately 10 dips and then counterstained in eosin-phloxine solution for 30 seconds to 1 minute. Last, slides were dehydrated and mounted with a xylene-based mounting medium. For Alcian Blue Staining, slides were stained for goblet cells and mucins using an Alcian Blue dye and Nuclear Red Fast counterstain kit (Abcam, Boston, MA, ab150662). Quantification of the percent area of Alcian blue staining, which calculated the percent area of the Alcian Blue dye compared with the rest of the tissue section, was calculated using the Alcian Blue color deconvolution plugin in ImageJ^28^ (ImageJ 1.53k Java 1.8.0_172).

### Immunofluorescent Staining (PGP9.5and Avpr1a)

On day 21 post SAL or ZYM treatment, adult male mice (n = 5/condition) were overdosed with inhaled isoflurane followed by intracardiac perfusion of ice-cold HBSS. About 4.5 cm of the distal colon was collected and post-fixed in 10% neutralized formalin fixative. After fixation, the distal colon tissue was embedded, cut, mounted, and washed as described above. Afterward, formalin-fixed tissue sections were deparaffinized and rehydrated. Blocking solution (5% donkey serum in phosphate-buffered saline solution + .1% Tween20 phosphate buffered saline with 0.1% Tween20 [PBST]) was applied for 20 minutes at room temperature. Primary antibodies were applied in blocking solution overnight in a humidified, light-protected chamber at 4 °C. A 1:15,00 anti-PGP9.5 (Catalog # 14730-1-AP, Proteintech, Rosemont, IL) and 1:1,000 anti-*Avpr1a* (Catalog # 720289, ThermoFisher Scientific, Waltham, MA) antibodies were applied. The next day, slides were washed with PBST for 15 minutes each (3 times). A secondary antibody cocktail was then applied (donkey anti-rabbit 555-red—PGP9.5 and donkey anti-mouse 488-green—*Avpr1a* in PBST) for 1 hour in a light-protected chamber at room temperature. Tissue sections were then washed for 15 minutes each time (3 times). 4’,6-diamidino-2-phenylindole and Vectashield mounting media were applied and covered with a coverslip. Images were taken on an 80i fluorescent scope using the 4’,6-diamidino-2-phenylindole, Green Fluorescent Protein, and cyanine 3 filter settings. Images were merged and processed using ImageJ.^28^

### Gastrointestinal Transit Time and Colonic Transit Time

Experimental measures for colonic motility were based on previous experimental design.^29^ Mice (n = 10/condition) were lightly anesthetized with 2% isoflurane in oxygen. The mouse’s head was carefully hyper-extended to pull out the tongue with blunt forceps. In all, 100 μL of fluorescein-labeled dextran (FITC-dextran, 70,000 MW, Sigma-Aldrich) was administered by oral gavage using a 1.0-mL syringe. While remaining under inhaled 2% isoflurane anesthesia, a 2-mm glass ball was inserted 3 cm past the anus into the colon using a fistula probe (metal rod, 2-mm diameter). Immediately after removing the fistula probe, mice were placed in a transparent cage, and the colonic transit time (CTT) was measured from when the fistula probe was pulled out of the colon to when the glass ball was excreted. For the remaining 90-minute period, mice were given access to food and water. After 90 minutes, mice were euthanized, and the entire GI tract from stomach to rectum was removed. The entire tract was placed on a polystyrene pad to avoid excess stretching.

To determine gastrointestinal transit (GIT), total length of the GI tract from the esophagus to the distal rectum was measured and then transected into 15 prepared segments: the stomach (#1), 10 equally sized small-bowel segments (#2–11), cecum (#12), and 3 equally sized colonic segments (#13–15). Each section was luminally flushed with 1 mL of Krebs Henseleit Buffer solution, collected in 2-mL tubes. The tubes were centrifuged at 12,000 rcf for 5 minutes at room temperature. The clear supernatants were transferred to new 1.5-mL tubes and stored in the dark at 4 °C until analysis (can be stored for several days at 4 °C without significant loss of Fluorescein isothiocyanate signal). For more extended storage, include .09% natrium azide or store at < −18 °C. For analysis, 100 μL of each supernatant was pipetted in duplicate onto a black 96-well plate (Greiner Bio One, Frickenhausen, Germany). Duplicate 100-μL Krebs Henseleit Buffer served as blanks. The fluorescence/well was quantified at 494 nm (absorption)/521-nm (emission) wavelength. For GI transit, the geometric center of FITC-dextran distribution, the center of gravity for the distribution of the marker, was calculated by the following formula^29^:

$$\text{GC}=\sum \left(\%\text{of}\;\text{total}\;\text{fluorescent}\;\text{signal}\;\text{per}\;\text{segment}\;\times \;\text{segment}\;\text{number}\right)/100$$

### Stool Consistency Assay

Stool consistency can be used as a quantitative assessment of water content in fecal samples, allowing for objective determination of the presence of altered fecal consistency and altered bowel habits (ie, diarrhea and/or constipation). Four fecal pellets were collected (immediately after excretion) from each mouse (n = 5/condition) over the timespan of VH development. Fecal pellets were weighed to 4 decimal places, and then microwaved for 6 minutes. Following dehydration, the pellets were weighed again. The wet weight and dry weight were normalized by dividing each weight by the number of pellets. The difference between the normalized wet and dry weights was used in statistical analyses.

### Retrograde Labeling of Colon-Specific Afferents

Fourteen days after treatment with ZYM (or SAL), mice (n = 5/condition) were anesthetized by inhaled isoflurane (4% for induction, 2% for maintenance), and a laparotomy was performed to open the abdominal cavity. Using a Hamilton microsyringe with a 22-gauge needle, 3 to 5 injections (2 mg/mL) of Alexa Fluor 488-conjugated cholera toxin-β (CTB) (Invitrogen, C-34775) were made beneath the serosal layer in the distal region of the colon. The total injection volume was 15 μL per mouse. After CTB injection, the abdominal incision was sutured and mice were returned to their home cage for 7 days before further experiments.

### Avpr1a mRNA Expression in Mouse Colon and Colon-Specific Extrinsic Primary Afferents

Mice of both substrains (n = 5/condition) were treated with either intrarectal SAL or ZYM for 3 consecutive days, as described above. Fourteen days after the final ZYM instillation, *retrograde labeling of colon-specific afferents* using CTB was performed, as described above.

#### Tissue Collection

Seven days later, mice were overdosed with isoflurane and perfused with ice-cold Ca^2+^/Mg^2+^ -free HBSS (Invitrogen, Waltham, MA). A 2 cm segment of the distal colon was collected and flash-frozen on dry ice. At the same time, thoracolumbar (T12-L2) and lumbosacral (L5-S1) dorsal root ganglia (DRG) were rapidly dissected and prepared for culture as described previously.^30^

#### Cellular Preparation

Dissociated cells were resuspended in F12 Nutrition Mixture (Invitrogen, REF# 11765-054) containing 10% fetal calf serum and antibiotics (penicillin/streptomycin, 50 U/mL). Dissociated cells were plated onto 12-mm laminin (.1 mg/mL) and poly-d-lysine- (5 mg/mL) coated glass coverslips (Corning Inc, Glendale, AZ). No additional growth factors were added to the culture medium. Cells were incubated overnight at 37 °C. Individual CTB-positive colon-specific neurons were collected with large-bore (~50-μm) glass pipettes and expelled into microcentrifuge tubes containing reverse-transcriptase (RT) mix (Invitrogen). All backlabeled neurons for a given mouse were pooled into separate tubes for lumbosacral and thoracolumbar DRG. For each experiment, negative controls consisted of omitting RT or using a cell-free bath aspirate as a template. The first-strand complementary DNA (cDNA) from a colonic sensory neuron was used as a template in a polymerase chain reaction (PCR) containing 1×*GoTaq* reaction buffer (Promega, Madison, WI), 20 mM outer primers, .2 mM deoxynucleotide triphosphates, and .2 mL *GoTaq* DNA polymerase (Promega). Each initial PCR product served as a template in a subsequent PCR reaction using a nested primer pair, the products of which were electrophoresed on 2% agarose-ethidium bromide gels and photographed. Only neurons producing detectable amplification of a housekeeping gene Glyceraldehyde 3-phosphate dehydrogenase (*Gapdh*) were analyzed further. The following primers were used for first-round amplification: *Avpr1a*, 5′-CCTACATGCTGGTGGTGATG-3′ (forward) and 5′-TCTTCACTGTGCGGATCTTG-3′ (reverse); *Gapdh*, 5′-AACTTTGGCATTGTGGAAGG-3′ (forward) and 5′-CCCTGTTGCTGTAGCCGTAT-3′ (reverse). For quantitative real-time PCR (qRT-PCR), the first-strand cDNA of cells expressing the target genes was pre-amplified (26 cycles) using the PCR condition as described above. For second-round (nested) amplification, the forward and reverse primer sequences were as follows: *Avpr1a*, 5′-GTCCGAGGGAAGACAGCATC-3′ (forward) and 5′-GATCTTGGCACGGGAAATGC-3′ (reverse); *Gapdh*, 5′-ATGAATACGGCTACAGCAACAGG-3′ (forward) and 5′-CTCTTGCTCAGTGTCCTTGCTG-3′ (reverse). The final amplification products had a predicted size of 338 bp (*Avpr1a*) and 301 bp (*Gapdh*) and were separated by electrophoresis. To ensure that observed differences in messenger RNA expression were not the result of differences in primer recognition of the cDNA template between strains (eg, due to unknown splicing variations, etc), all primers underwent a multistep validation process. First, we determined that our primers were equally efficient in both strains using different ratios of cDNA and primer volumes added to each well as described in the Pfaffl method.^31^ All wells received the same amount of SYBR green and total reaction volume, while primer and sample concentrations were varied on a logarithmic scale. Pilot samples from both strains of mice were run through 40 cycles of PCR to confirm that their efficiency for PCR product doubling at each cycle was 90% less in both strains and then the products underwent melt curve analysis to confirm a single product in the well (either target or housekeeping as appropriate). All samples were then run on a 2% agarose gel and imaged using BioRad ChemiDoc MP Imaging System to confirm a single band representing a single product.

The products were used as a template for qRT-PCR using ABsolute QPCR SYBR Green ROX mix (ABgene, Rochester, NY) in an Applied Biosystems (Foster City, CA) 5700 real-time thermal cycler. Colorectal samples only underwent PCR with the second-strand (nested) primer set, as the preamplification of the target product was not necessary for adequate quantification. Threshold cycle (C_t_) values were recorded to measure initial template concentration, and relative fold changes in messenger RNA (mRNA) levels were calculated using the 2^−ΔΔCt^ method using *Gapdh* as a reference standard.

### Colorectal Avpr1a Protein Expression

Total protein was isolated from approximately 50 mg of snap-frozen distal colon tissue using Cell Extraction Buffer (Invitrogen) containing Halt protease and phosphatase inhibitors (ThermoFisher Scientific) and Na_3_VO_4_. Protein concentrations were determined using the Nanodrop spectrophotometer (Thermofisher Scientific, Waltham, MA). Protein levels of *Avpr1a* (pg/mL) were measured using ELISA per the manufacturer’s instruction (MBS, Cat# MBS2502155, San Diego, CA).

### Mechanical Response of Colorectal Tissue Strips to Stretch

We followed a previously described procedure to measure the force-displacement relations with slight modifications.^32^ Briefly, mice (n = 4–6/condition) were killed via CO_2_ inhalation followed by exsanguination after perforating the right atrium. The distal colon was dissected and transferred to ice-cold Krebs solution bubbled with carbogen (95% O_2_, 5% CO_2_). The colon was opened longitudinally, pinned flat mucosal side up in a tissue chamber. Rectangular strips measuring 8 mm × 4 mm were cut along the longitudinal (long) or circumferential (circ) directions and transferred to the tissue testing chamber for axial stretch testing. The testing chamber was superfused with a modified Krebs solution, at room temperature (approximately 30 °C), containing the following (in mM): 117.9 NaCl, 4.7 KCl, 25 NaHCO_3_, 1.3 NaH_2_PO_4_, 1.2 MgSO_4_, 7 H_2_O, 2.5 CaCl_2_, 11.1 D-glucose, 2 butyrate, and 20 acetate. One end of the tissue strip was pinned down and the other end tied via silk suture thread (size 6.0) to a dual-mode servo actuator for force and displacement measurement with a force precision of .1 mN and a displacement precision of .1 mm (Model 300D, Aurora Scientific, Aurora, CA). Data were digitized and recorded using a data acquisition system (1401Plus, CED, UK) and Spike2 software (v7.0, CED, UK). To assess the effect of arginine-vasopressin (AVP) on the mechanical response of the colorectal tissue, we implemented a slow ramped stretch protocol, including symmetric loading and unloading force ramps from 0 to 45 mN at 1.5 mN/s. Prior to testing, we preconditioned the tissue by repeating the stretch protocol 10 times to achieve a stable and repeatable recording of the force-displacement relation. Following baseline force-displacement recording, AVP (.1 μM) was added to the bath solution for 5 minutes, and the force-displacement measurement was repeated. Subsequently, the bath solution was replaced with regular Krebs solution for a 30-minute washout period before conducting another force-displacement measurement. Force-displacement data were quantified as the AUC and normalized to the baseline response.

### Dissociation, Culture, and Ca^2+^ Imaging

Seven days after injection of retrograde tracer, mice were overdosed with inhaled isoflurane (> 5%) and transcardially perfused with 4 °C Ca^2+^/Mg^2+^-free 1× HBSS (Invitrogen). Bilateral thoracolumbar DRGs, LS DRGs, or nodose (ND) ganglia were dissected and placed separately into 1× HBSS for cellular dissociation. The enteric nervous system (ENS) was collected from a separate cohort of mice. The myenteric plexus was collected and dissociated as described elsewhere.^33,34^ Briefly, 1 to 2 cm of the distal colon was collected per mouse and placed in ice-cold 1× HBSS. Colon samples were mounted to an agar plate surface (SYLGARD 170 Silicone Elastomer Kit) while submerged in 1× HBSS. Additional mesenteries, fat tissue, and fecal matter were removed. The mucosa layer, followed by submucosal layer, was carefully removed to easily isolate enteric neurons from the myenteric plexus. Isolated myenteric tissue was placed in a culture tube for cellular dissociation. Enzymatic dissociation of smooth muscle and myenteric plexus tissue was performed as described in previous work.^33,34^ After cellular dissociation of each tissue sample (DRG, ND, Enteric) (as stated above), cells were resuspended in F12 media and plated onto 12-mm poly-D-lysine/laminin precoated coverslips (refer to *Avpr1a* mRNA Expression in Mouse Colon and Colon-Specific Extrinsic Primary Afferents above for products information). Cells were incubated overnight at 37 °C and imaged the following day (16–24 hours after plating cells) as published elsewhere.^35^ Before imaging, each coverslip was incubated in 3 μL of fura-2 acetoxymethyl ester (Invitrogen) in 1× HBSS containing 5 mg/mL Bovine Serum Albumin (Sigma-Aldrich) for 30 minutes at 37 °C. Coverslips were placed in a Quick Exchange −1 quick change platform (Warner Instruments, Hamden, CT) and mounted on a Nikon Eclipse Ti-inverted microscope (Nikon, Melville, NY BioRad ChemiDoc MP Imaging System, BioRad, Hercules, CA) stage. Coverslips were exposed to a constant flow of 1× HBSS buffer that was controlled by a gravity flow system (Warner Instruments). Perfusate temperature was maintained at 30 °C, and all agonists were dispensed with a gravity-feed pinch valve control perfusion system and included 30 mM K^+^ (high K^+^),^35^ capsaicin (Sigma-Aldrich, 1 μM), and AVP (Millipore Sigma, Burlington, MA, V9879, .1 μM). Capsaicin was dissolved in 1-methyl-2-pyrrolidinone to create a 10-mm stock solution; 1.0 μM capsaicin was made fresh daily in 1× HBSS. Firmly attached, CTB-positive neurons were identified as regions of interest in the software (Nis-Elements Version 4.60, Nikon, Melville, NY). Unlabeled, adjacent cells were also identified and imaged. Absorbance data at 340 nm and 380 nm were collected at 1 Hz during drug application using a pco.Edge 4.2 LT camera (PCO-TECH, Romulus, MI). Responses were measured as the ratio of 340/380 nM excitation (ΔF_340/380_) and 510-nm emission controlled by a high-speed Fura/wide-field xenon-illuminated filter wheel (Boyce Scientific, Gray Summit, MO). All fields were first tested with a brief application (5 seconds) of high K^+^ and only cells responsive to high K^+^ were identified as healthy and responsive and included in subsequent analyses. Five minutes after application of high K^+^, capsaicin was applied until cells started to show peak fluorescence responses (typically ~2 seconds after the application start), followed by a 10-minute washout period prior to AVP application. AVP was applied via a micropipette in droplet intervals until cells started to show peak fluorescence responses (typically shown immediately after a couple of droplets are placed onto the coverslip). Once an increased change in fluorescence occurred, the agonist application of each agonist is stopped, and a wash buffer, 1× HBSS, is turned on for approximately 5 seconds. Peak Ca^2+^ influx was calculated using MATLAB (Mathworks, Natick, MA), and responses > .1 ΔF_340/380_ were included in the analysis. The prevalence of capsaicin- or AVP-responsive neurons was determined as a percentage of total healthy (high K^+^-responsive) CTB-positive and unlabeled cells for both DRG and ND ganglia. All responsive enteric neurons were included in the analyses.

### Clinical Subject Recruitment, Phenotyping, and Sample Collection

The methods for sample collection and recruitment of ulcerative colitis (UC) patients have been described in detail elsewhere.^36^ Individuals with an established diagnosis of UC and healthy controls undergoing colonoscopy at a large, metropolitan, academic medical center, were enrolled. All participants rated abdominal pain using a visual analog scale and completed standardized surveys addressing anxiety or depression (Hospital Anxiety and Depression Scale) and GI symptoms (Rome-III questionnaire). Patient age, sex, and severity of inflammation (based on endoscopic and histologic assessment) were determined. A subset of study participants used in a previously published study^36^ was divided into the following cohorts for analysis of *Avpr1a* mRNA expression (based on the underlying diagnosis and pain status): 1) inactive UC without (abdominal) pain, 2) active UC without (abdominal) pain, 2) inactive UC with (abdominal) pain, 4) active UC with (abdominal) pain. In addition to UC patient recruitment and sample collection at the University of Pittsburgh, we analyzed a subset of pediatric IBS patients undergoing colonoscopy at Connecticut Children’s Medical Center. Pediatric patients (males and females, age 7–17) undergoing diagnostic colorectal biopsy (n = 3/patient) for differential diagnosis of recurrent abdominal pain were compared to pediatric patients undergoing painless noncancerous polyp monitoring.^37^ Patients meeting Rome-III criteria for IBS (and controls) in the absence of other diagnoses (eg, *H pylori* and inflammatory bowel disease) or clinical signs of disease were administered the Pain Burden Inventory (PBI) to measure self-reported pain severity. We evaluated relative *Avpr1a* mRNA expression (2^−ΔΔCt^ method) in biopsies from all patients and compared IBS patients with low and high self-reported pain (defined as a PBI score of 0–11 [low pain, n = 10] or greater than a PBI score of 12–22 [high pain, n = 14], respectively). The definitions of low and high pain burden were empirically determined using the mean (11.71) of all PBI scores to define the 2 comparison groups, however, this is a different comparison than that described above for the UC dataset as the diagnosis of functional abdominal pain/IBS requires the presence of abdominal pain. All pain-free controls (n = 3) reported a PBI = 0.

### Assessment of Avpr1a mRNA Expression in Human Rectal Biopsies

To assess the translational potential of this relationship between *Avpr1a* expression and persistent abdominal pain, we evaluated *Avpr1a* mRNA expression in colorectal biopsies previously collected from 2 cohorts of patients with UC and IBS and comparing those with pain to those reporting no pain at the time of diagnosis.^36^ If *Avpr1a* expression contributes to persistent visceral VH/abdominal pain in humans, as in our mouse model, then expression should differ between patients with and without pain. Rectal biopsies were obtained and processed using qRT-PCR to determine the transcript expression of *Avpr1a* (PrimerBank ID: 33149325c1). Total RNA was extracted using RNeasy isolation columns (Qiagen, Hilden, Germany) and real-time quantitative PCR was undertaken as described previously.^38^ According to the manufacturer’s instructions, mRNA samples underwent reverse transcription with iScript cDNA synthesis kits (BioRad, Hercules, CA). Quantification with a nana-drop and loading of 2 ng of total cDNA were placed into each reaction. cDNA template was then used for qRT-PCR SSO Universal SYBR Green Master Mix with ROX (BioRad) in an Applied Biosystems StepOne Plus PCR machine. C_t_ values were recorded as a measure of initial template concentration, and relative fold changes in mRNA levels were calculated by the 2^ΔΔCt^ method using GAPDH as a reference standard: GAPDH, 5′-AAG-GAC-TCA-TGA-CCA-CAG-TTC-ATG-3′ (forward strand) and 5′-TTG-ATG-GTA-CAT-GAC-AAG-GTG-CGC-3′ (reverse strand). Primer sets were designed using primer-BLAST (Basic Local Alignment Search Tool, NCBI). All primer sets were evaluated for efficiency and were found to have > 95% amplification of the template sequence at 1 μM concentration. Single-sequence amplification was confirmed by running melt curve analysis on all PCR products, and all products underwent gel electrophoresis to confirm a single amplified product of the expected size. The following primers were used for *Avpr1a* amplification: *Avpr1a*, 5′-TGT-AAA-ACG-ACG-GCC-AGT-CCC-CAG-AGT-TAA-GAC-AGT-TGC-3′ (forward strand), and 5′-CAG-GAA-ACA-GCT-ATG-ACC-CCG-GTT-TAC-CCT-TGC-ACT-TT-3′ (reverse strand).

### Statistical Analysis

Outcomes from VMR were analyzed using a 2 (BL/6NTac vs BL/6J) × 2 (pre ZYM vs post ZYM-VH) repeated measure comparison. Post hoc group comparisons using Fisher’s Least significant difference confirmed that the VMR to 60-mmHg distention pressure differed between B/6NTac-ZYM and all other conditions RT-PCR, ELISA, single-fiber recordings, and Ca^2+^ imaging were analyzed using 2 (BL/6NTac vs BL/6J) × 2 (ZYM vs SAL) analysis of variance (ANOVA) followed by Bonferroni t-tests for critical post hoc comparisons. This is followed by both a Levene Test for Equality of Equal Variance and a Bonferroni post hoc for further statistical comparison. Histological stains were evaluated for the percent of colocalization with markers of other cell types (neuronal, muscle, etc) to determine the distribution of *Avpr1a* within the heterogeneous cell makeup of the colon wall using programs such as ImageJ/FIJI.^39^ For both clinical cohorts, fold differences in expression compared with the healthy control condition were analyzed using 1-way ANOVA (*P* < .05). All data are presented as mean ± standard error of the mean. For all reported data, the main effects and interactions are found in figures, and all significant post hoc comparisons are indicated by *.

## Results

### BL/6Substrains Exhibit Differential Susceptibility to VH Following ZYM Administration

Visceral sensitivity was first determined for BL/6J and BL/6NTac mice by measuring the VMR during colorectal distention before ZYM treatment and 21 days after the final ZYM treatment in the same cohort of mice. A total of 2 strains at 2 time points (before and 21 days after ZYM treatment) were thus compared: BL/6J-naïve (baseline), BL/6J-ZYM, BL/6NTac-naïve (baseline), and BL/6NTac-ZYM. 2 (BL/6NTac vs BL/6J) × 2 (pre- vs post ZYM exposure) ANOVA on the cumulative AUC across all distension pressures revealed a significant main effect of strain and a significant Strain × Time interaction. This effect appeared to be primarily driven by hypersensitivity to CRD at the highest distension pressure (Fig 1). One-way ANOVA considering only VMR to 60-mmHg distention pressure confirmed a significant main effect of the condition (*P* < .05). Post hoc group comparisons using Fisher’s Least significant difference also confirmed that the VMR to 60-mmHg distention pressure differed between B/6NTac-ZYM and all other conditions (all *P* < .05). No other significant differences were present. Refer to Supplementary Fig 1 for representative EMG recordings between VH-resistant C57BL/6J and VH-susceptible C57BL/6NTac at 60 mmHg before and 21 days after ZYM.

### ZYM Treatment Decreases CTT and Increases Fecal Water Retention, but Does Not Affect GIT or Body Weight in BL/6Substrains

**Figure 2.**
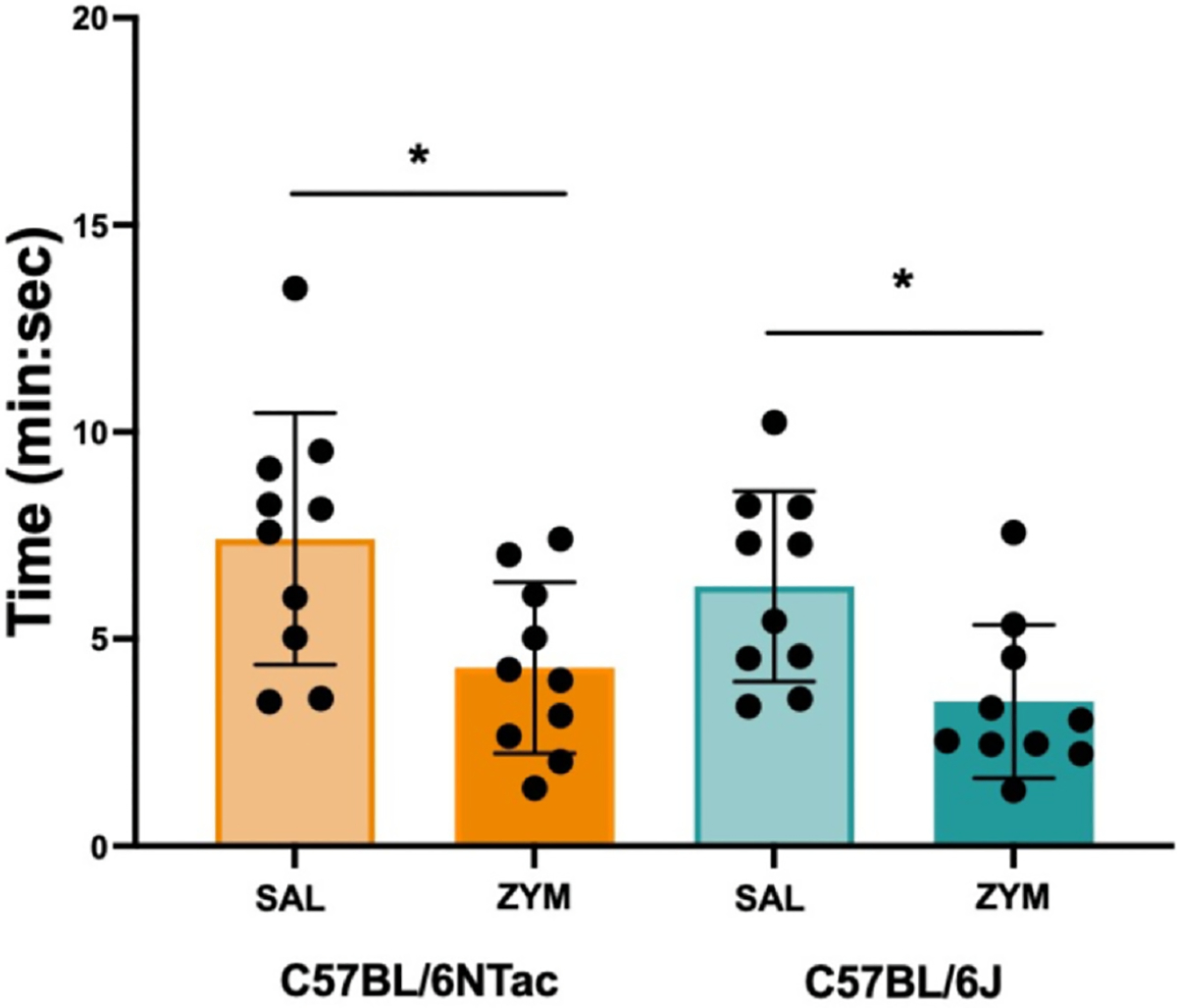
ZYM treatment increases CTT in both C57BL/6 substrains. CTT was measured in SAL-treated BL/6J (light teal), ZYM-treated BL/6J (dark teal), SAL-treated BL/6NTac (light orange), and ZYM-treated BL/6NTac mice (dark orange) 21 days after treatment when VH has developed. A 2 × 2 ANOVA revealed a significant main effect of ZYM treatment regardless of substrain (F [1, 36] = 13.336, *P* < .001). No other significant main effects or interactions were observed (all F’s ≤ 1.219, all *P* > .05) (n = 10). * Indicates significant main effect of ZYM from ANOVA, *P* < .05.

**Figure 3.**
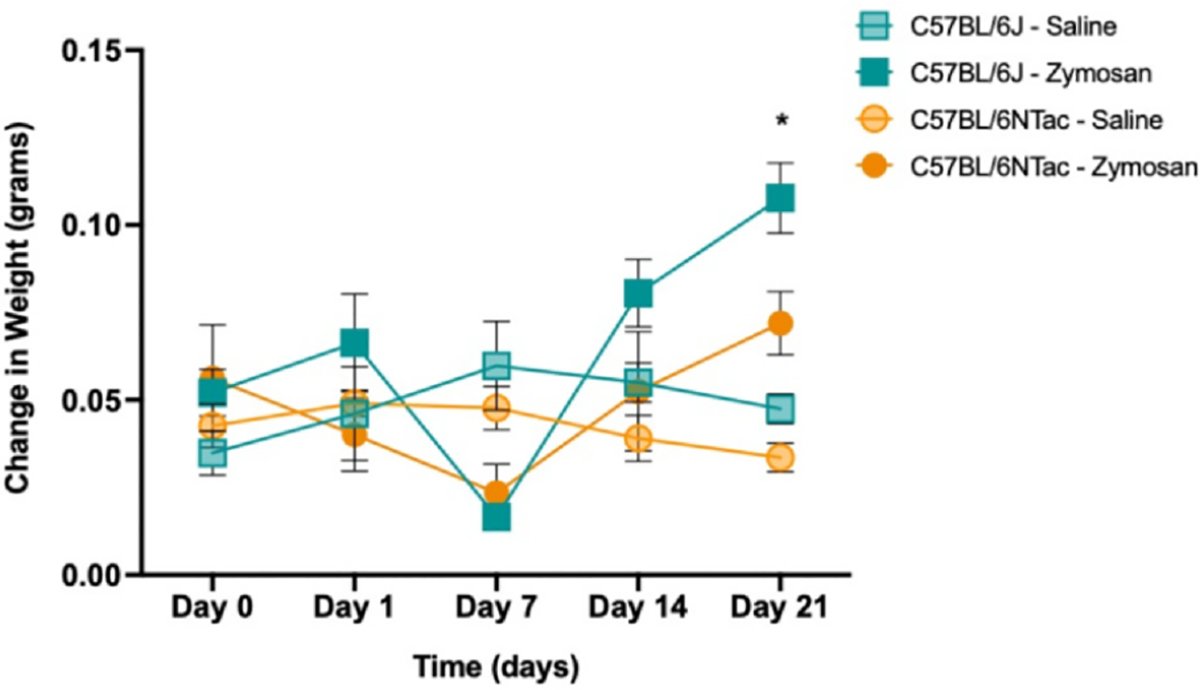
ZYM treatment increases fecal water retention at D21 regardless of strain. Change in fecal water weight was measured by collecting fecal weight (4 pellets collected/mouse) at 2 time points: 1) immediately after a mouse excreted pellets (wet weight) and 2) after fecal matter had dried (dry weight) throughout VH development. Average weight differences (wet fecal matter − dry fecal matter) for each strain and condition are shown above. BL/6J-SAL = light teal square; BL/6J-ZYM = dark teal square; BL/6NTac-SAL = light orange circle; BL/6NTac-ZYM = dark orange circle. 2 × 2 repeated measures ANOVA revealed a significant main effect of time (F [4, 64] = 2.625, *P* = .043) and a Time × Condition interaction (F [4, 64] = 6.834, *P* < .001) but no other significant main effects or interactions (F’s ≤ 3.235, all *P* > .05). A follow-up 2 × 2 ANOVA conducted on D21 data only revealed a significant main effect of condition (F [4, 64] = 2.635, *P* = .007), indicating that 21 days after ZYM instillation there is a co-occurring increase in fecal water retention regardless of strain (n = 5).

No significant main effects or interactions were observed for GIT (Supplementary Fig 2) when comparing substrains (BL/6J and BL/6NTac) or treatment conditions (ZYM and SAL) (all *P* > .05). However, we identified a significant main effect of treatment, where ZYM instillation resulted in decreased CTT regardless of strain (Fig 2), indicating that BL/6J mice exhibit dysmotility, while BL/6NTac mice exhibit co-occurring dysmotility and VH (*P* < .05). Decreased CTT means that movement through the colon is more rapid and may reduce the water absorption from fecal boli resulting in diarrhea. In line with this finding, we observed significant differences in stool consistency (fecal water retention) in both BL/6J and BL/6NTac mice at 21 days after ZYM treatment (at VH development) (*P* < .05) (Fig 3). ZYM causing low-level diarrhea could be clinically relevant, so we measured body weight to assess dehydration and calorie absorption that could be affected. No significant differences in body weight were found between strains or as a result of ZYM treatment at any time during the development period for VH (all *P* > .05, Supplementary Fig 3). While outside the scope of the current study, this finding suggests that ZYM treatment in BL/6J mice may represent a model for colonic dysmotility in the absence of pain as seen in idiopathic/functional diarrhea.

### Positional Identification of Avpr1a as a VH Candidate Gene

**Figure 4.**
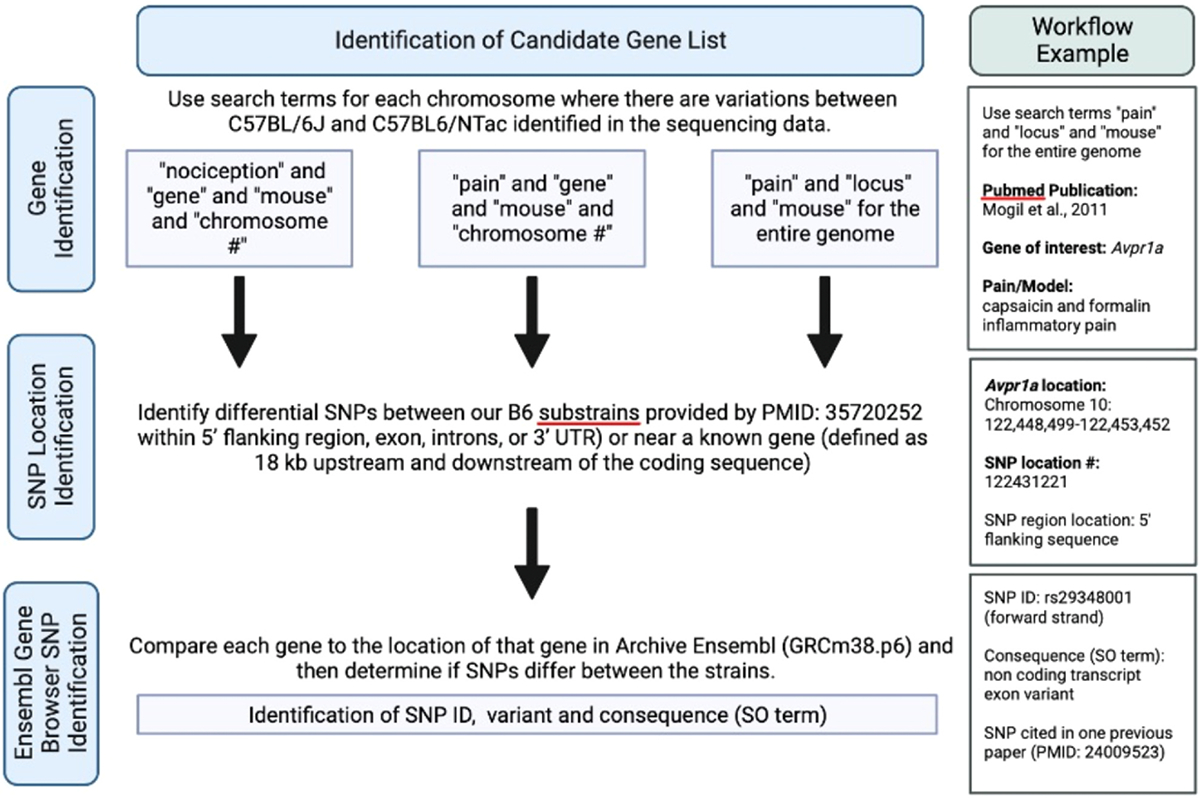
Workflow for identifying pain candidate genes from whole-genome sequencing data. In brief, we performed a systematic review and collected a list of genes related to visceral pain, nociception, and/or pain assays. From this list, we used whole-genome sequencing data published in Mortazavi et al^21^ to compare the location of our genes of interest to locations of possible differential SNPs between BL/6J and BL/6NTac. It is important to note Archive Ensembl was used to match and compare whole-genome sequencing data to the appropriate gene loci. To the right of the general flow is an example workflow used to identify an intergenic SNP within the 5′ region of *Avpr1a*.

Various phenotypic differences have been reported among BL/6 substrains with findings related to learning and memory performance, glucose metabolism, and drug/alcohol responses.^40,41^ Prior work using low-resolution SNP genotyping^42^ identified 12 (out of 1,449) SNPs differed between BL/6J and BL/6NTac mice.^23^ Of these 12 SNPs, 3 were located on distal chromosome 10, a region previously identified in 2 separate QTL for inflammatory pain sensitivity using an injection of formalin into the hind paw^43^ and peritoneal acetic acid administration.^44^ These 2 QTL converge on a single-candidate gene from chromosome 10, *Avpr1a*, which has subsequently been validated/confirmed as contributing to differences in somatic pain sensitivity in both human and animal studies.^45^ Extending this promising finding,^42^ we leveraged recently published whole-genome sequencing of SNVs between BL/6 substrains^21^ and identified 5 genes where SNVs differentiated the VH-susceptible and VH-resistant BL/6 substrains (refer to Fig 4 for a diagram of our gene database workflow). Of these 5 candidates, only *Avpr1a* was associated with variability in more than 1 pain assay or measure (Table 1). We identified a SNV in the 5′ flanking sequence for *Avpr1a*, a region heavily implicated in regulating *Avpr1a* expression.^43,44^ Moreover, we found that this SNV represents an exon variant within an uncharacterized long intergenic noncoding RNA, further implicating a regulatory role for this variant. As such, *Avpr1a* was identified as our highest-priority candidate gene for VH.

### Increased Avpr1a Expression in the Colorectum but Not Colon-Specific Extrinsic Primary Sensory Neurons Corresponds to VH

**Figure 5.**
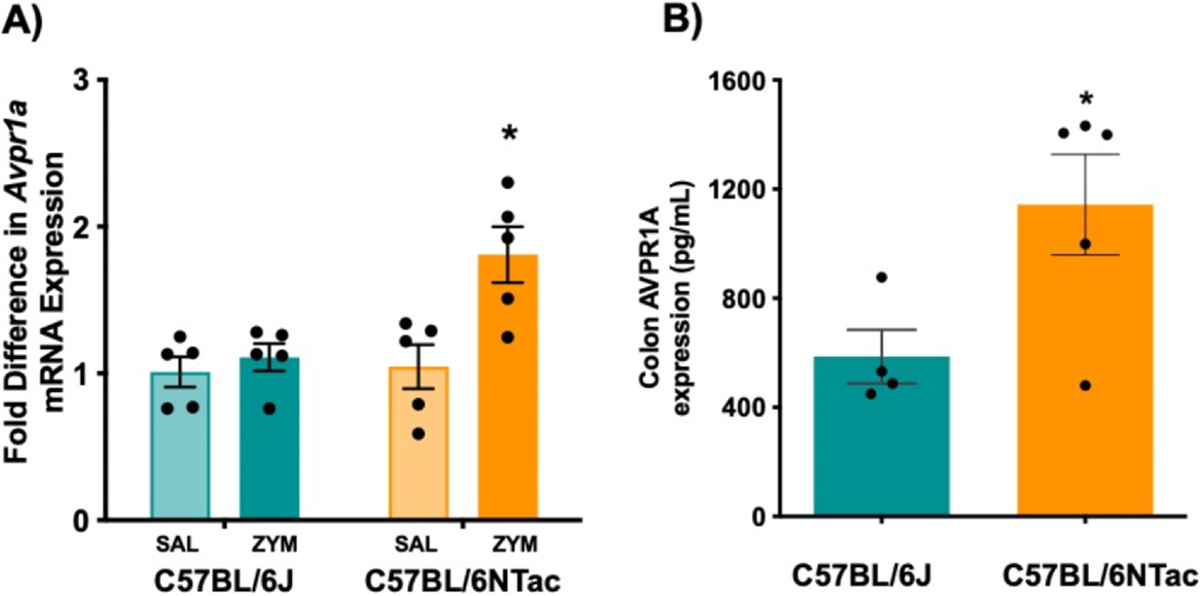
Colonic *Avpr1a* expression is greater after ZYM exposure in C57BL/6NTac mice, but not C57BL/6J mice. (**A**) *Avpr1a* mRNA levels were measured in SAL or ZYM-treated BL/6J and BL/6NTac mice. A 2 (strain) × 2 (treatment) ANOVA confirmed a significant Strain × Treatment interaction (F [1, 16] = 5.664, *P* = .030) on colorectal *Avpr1a* expression. * Indicates significant interaction based on ANOVA, *P* < .05. (**B**) *Avpr1a* protein levels in isolated colons were higher in ZYM-treated BL/6NTac compared with ZYM-treated BL/6J mice. * Indicates significant independent samples t-test (t = 2.669, *P* < .05) (n = 5).

*Avpr1a* mRNA expression was determined in both homogenized colorectum and colon-specific primary sensory neurons from BL/6J and BL/6NTac mice, 21 days after SAL or ZYM treatment. *Avpr1a* mRNA expression was increased in the colorectum (Fig 5A) but not in colon-specific DRG neurons in BL/6NTac mice treated with ZYM compared with all other conditions. No significant main effects or interactions on the expression of *Avpr1a* in either colon-specific thoracolumbar or lumbosacral primary sensory afferents were detected in a similar 2 × 2 ANOVA (Supplementary Fig 4). In agreement with increased *Avpr1a* mRNA expression, colorectal *Avpr1a* protein levels were significantly higher in BL/6NTac mice compared with BL/6J mice 21 days after ZYM (*P* < .05) (Fig 5B).

### Distal Colorectal Morphology (Inflammation, Goblet Cell, and Mucus Production) Does Not Differ Between Strains or as a Result of ZYM Treatment

Hematoxylin and eosin staining was performed to detect the presence of inflammatory cells. No significant differences between strains or conditions at day 21 post ZYM/SAL installation, in line with previous reports that the neutrophil-based inflammation induced by ZYM instillation resolves quickly prior to the emergence of VH.^46^ Representative data are presented in Supplementary Fig 5. Alcian Blue staining, which visualizes acidic epithelial and connective tissue mucins, allowing the measurement of the percent surface area of mucin cell bodies,^47^ reveals no changes in percent mucin area regardless of strain or condition (all *P* > .05) (Supplementary Fig 6), indicating that the mucus-producing cells are present and functional in both strains after ZYM.

### Functional Sensitivity to Stretch in the Presence of the Avpr1a Agonist AVP is Greater in BL/6NTac Mice Treated With ZYM

**Figure 6.**
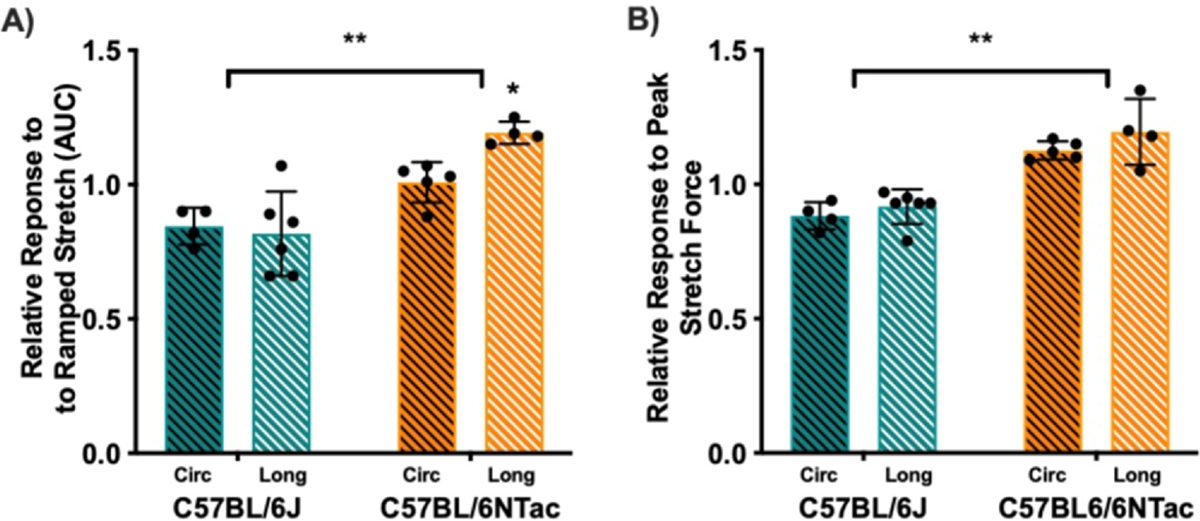
Force-displacement relation in response to circumferential (circ, black stripes) and longitudinal (long, white stripes) stretch is increased in C57BL/6NTac mice in the presence of AVP. In ZYM-treated BL/6J (teal bars) and BL/6NTac (orange bars) mice, the force-displacement relation was measured before and after AVP application in the bath solution. Data are presented as AUC and peak responses to stretch during AVP application relative to the baseline measures. (**A**) A 2 (Strain) × 2 (Stretch Direction) ANOVA revealed a significant main effect of Strain (F [1, 15] = 30.810, *P* < .001) and a Strain × Stretch Direction interaction (F [1, 15] = 4.787, *P* < .05) on AUC. (**B**) Similarly, 2 × 2 ANOVA for force-displacement during peak stretch force application confirmed a significant main effect of Strain (F [1, 15] = 57.203, *P* < .001), confirming a greater response magnitude in BL/6NTac mice treated with ZYM compared with BL/6J mice treated with ZYM though this did not differ for the 2 stretch directions. No other significant main effects or interactions were observed (all *P* > .05) (n = 4–6). * indicates significant strain x stretch direction interaction based on ANOVA. ** indicates significant main effect of strain.

Both AUC for the force-displacement relation during ramped stretch and response magnitude to peak stretch force (45 mN) were recorded before and after the application of AVP (Fig 6). Specifically, BL/6NTac-ZYM mice had greater AUC compared with BL/6J-ZYM mice, and longitudinal stretch in BL/6NTac-ZYM mice elicited the greatest response magnitude (greatest AUC) compared with all other groups (*P* < .05) (Fig 6A). A 2 (Strain) × 2 (Stretch Direction) ANOVA revealed a significant main effect of Strain, and a Strain × Stretch Direction interaction (all *P* < .05). 2 × 2 ANOVA on force-displacement during peak stretch force application confirmed a significant main effect of Strain (*P* < .001), confirming a greater response magnitude in BL/6NTac mice treated with ZYM compared with BL/6J mice treated with ZYM though this did not differ for the 2 stretch directions (Fig 6B). No other significant main effects or interactions were observed (all *P* > .05). Still, these data align with the strain difference in susceptibility to the VH phenotype even though the maximum stretch force used here corresponds to lower CRD pressure than those administered when measuring VH by VMR to CRD. These data confirm that the increased *Avpr1a*/*Avpr1a* expression has functional consequences for the response to colorectal stretch corresponding to increased expression and behavioral hypersensitivity in BL/6NTac mice.

### Immunohistochemistry Staining Reveals Possible Colocalization Between Avpr1a and Neuronal Terminal Endings

**Figure 7.**
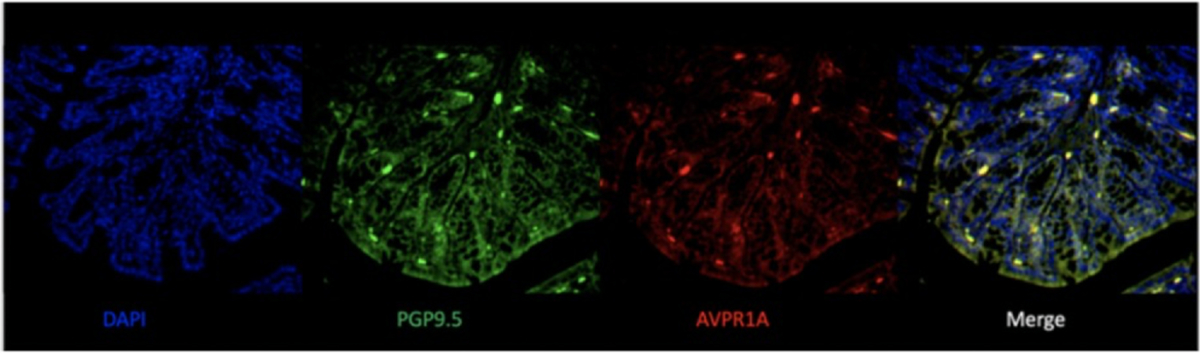
Representative images of immunohistochemistry stains of distal colon cross sections from VH mice show colocalization of *Avpr1a* and neurons. Distal colon samples from BL/6NTac mice were collected at VH development. Sections were stained with DAPI nuclear (blue), PGP9.5 for neurons (green), and *Avpr1a* (red). Images were merged from all 3 to show colocalization of *Avpr1a* and neuronal cell bodies (yellow). Abbreviations: DAPI, 4’,6-diamidino-2-phenylindole.

Colabeling of the distal colon with antisera against PGP9.5 (neuronal marker) and *Avpr1a* revealed overlap of the 2 markers, suggesting the possibility that *Avpr1a* is expressed in neurons with terminals in the colon wall (Fig 7). PGP9.5 labeling does not discriminate between extrinsic primary afferents (DRG neurons) and neurons of the ENS, but we have already shown that ZYM-induced increases in *Avpr1a/Avpr1a* were restricted to colonic tissues (refer to Fig 4) but not in colon-specific DRG neurons (refer to Supplementary Fig 4).

### In Vitro Ca^2+^ Imaging of ENS Neurons From VH Mice Exhibited Significantly Greater Ca^2+^ Influx to AVP

**Figure 8.**
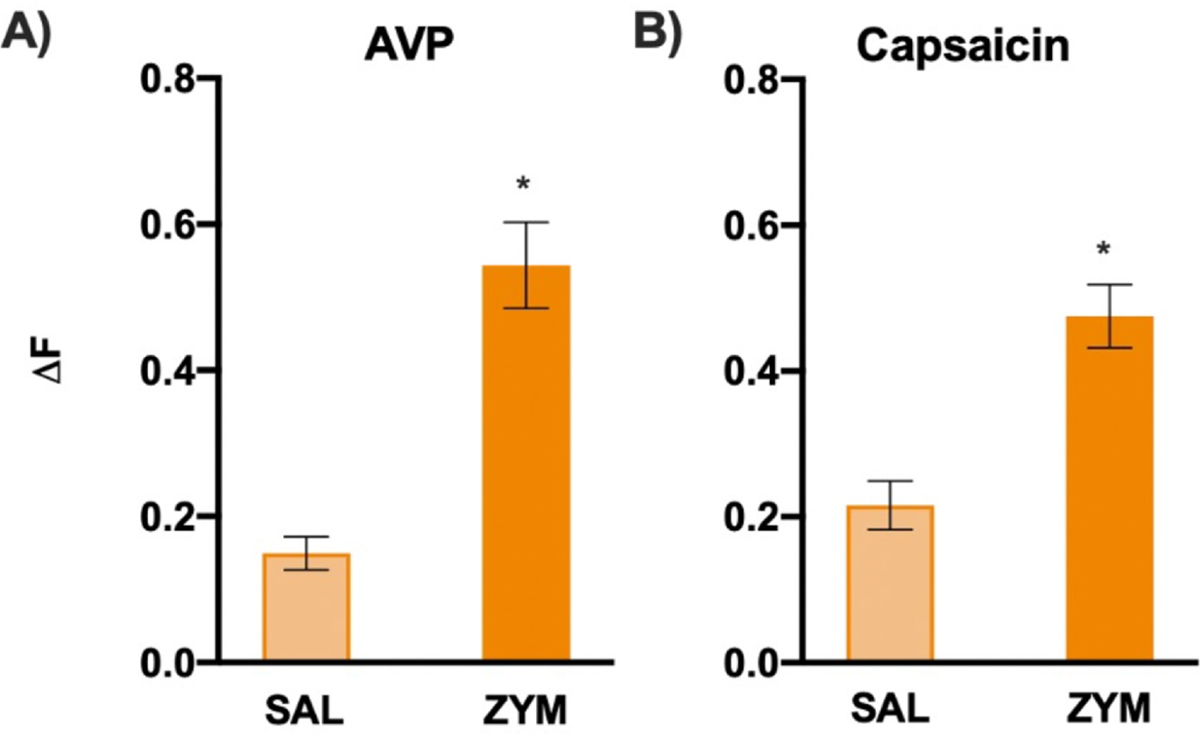
In vitro Ca^2+^ imaging of ENS neurons from VH mice exhibits significantly greater Ca^2+^ influx to AVP and capsaicin. (**A**) In vitro Ca^2+^ imaging of ENS neurons revealed individual neurons from VH mice (BL/6NTac-ZYM) exhibited significantly greater Ca^2+^ influx (ΔF) in response to AVP compared with SAL controls (t = −4.556, *P* < .001). Refer to Supplementary Fig 8 for individual ΔF distributions for each condition and agonist. (**B**) ENS neurons revealed individual neurons from VH mice also exhibited greater Ca^2+^ influx (ΔF) in response to capsaicin compared with SAL controls (t = −4.838, *P* < .001). The % of neurons responding to AVP and capsaicin did not differ significantly (data not shown) (all *P* > .05). Refer to Supplementary Fig 9 for representative traces of enteric neuron responses for each condition and agonist. * Indicates significant t-test, *P* < .05.

Ca^2+^ transients in separate cultures of extrinsic colon-specific DRG and enteric neurons were measured in response to 1) the *Avpr1a* agonist, AVP; and 2) the TRPV1 agonist capsaicin. When comparing neurons from BL/6NTac mice with ZYM-VH to SAL-treated controls, neither DRG nor ND ganglion neurons retrogradely labeled from the colon exhibited altered cellular Ca^2+^ influx (ΔF_340/380_, presented as ΔF in figures) (Supplementary Fig 7) or changes in the % of cells responding to any chemical stimulus (data not shown) (all *P* > .05). Enteric neurons isolated from the myenteric plexus exhibited increased responses (ΔF) to AVP (Fig 8A) and capsaicin (Fig 8B) (all *P* < .001), suggesting enteric neuron hyperresponsiveness may play a role in amplification of sensory transmission during mechanical distension and the development of VH in BL/6NTac treated with ZYM. Refer to Supplementary Fig 8 for individual enteric neuron responses (ΔF) to AVP and CAP. Refer to Supplementary Fig 9 for representative traces of individual enteric neurons from each condition and agonist.

### Avpr1a/Avpr1a Expression in Colorectal Biopsies From Clinical Cohorts With and Without Persistent Abdominal Pain and Healthy Controls

**Figure 9.**
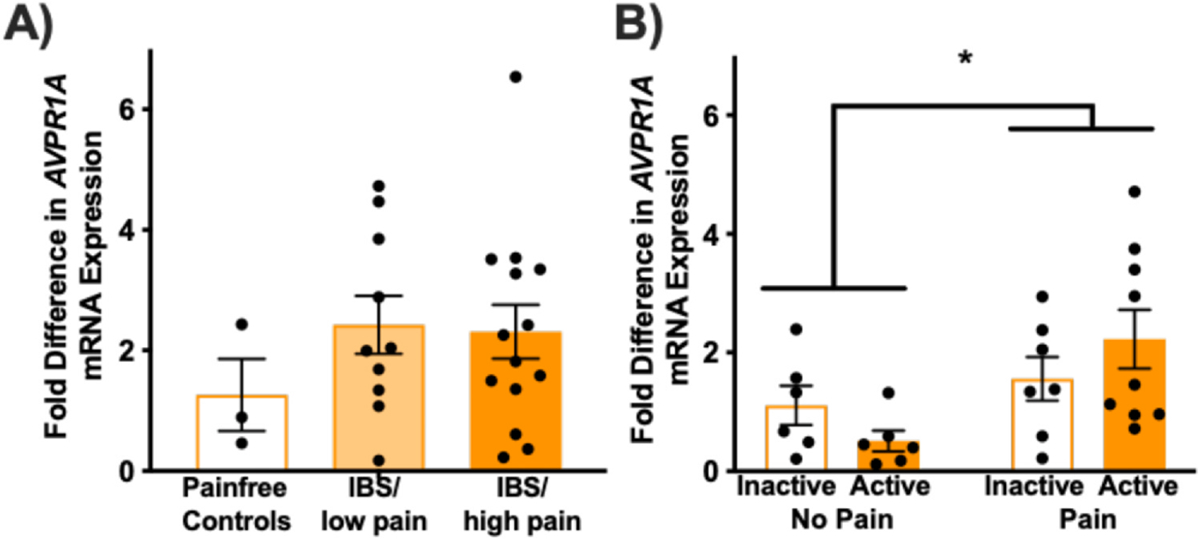
*Avpr1a* expression is increased in colorectal biopsies from clinical cohorts with abdominal pain. (**A**) *Avpr1a mRNA* levels from pediatric IBS patients’ colonic biopsies with low or high levels of reported pain compared with pain-free controls. One-way ANOVA showed no significant differences between conditions and follow-up planned comparisons with independent samples t-tests confirmed that there were no differences between IBS/low pain (n = 10) or IBS/high pain (n = 14) compared with pain-free controls (n = 3) (all *P* > .05). This could be due to a low number of controls and difficulty finding appropriate controls given that preventative colonoscopy is not recommended until adulthood and these are all adolescents. (**B**) *Avpr1a mRNA* levels from adult patients with active or inactive UC who report pain compared to those reporting no pain. n = 6 to 9 per group. A 2 (Active vs Inactive) × 2 (Pain vs No Pain) ANOVA confirmed a main effect of pain status (F [1, 24] = 6.935, *P* < .05) but not disease status. * No other significant main effects or interactions were present (all F ≤ 2.396, all *P* > .05). * Indicates significant ANOVA, *P* < .05.

In support of the experimental data described above, the clinical evaluation of *Avpr1a* expression was explored in 2 cohorts of patients with varying self-reported intensities of abdominal pain. In Fig 9A, pediatric patients (males and females, age 7–17) undergoing diagnostic colorectal biopsy for abdominal pain were compared with pediatric patients undergoing colonoscopy for functional nausea or painless noncancerous polyp monitoring (n = 3 patients). Patients with Rome-III criteria for IBS (and controls) in the absence of other diagnoses (eg, active *Helicobacter pylori* infection and/or inflammatory bowel disease) or clinical signs of disease were assessed using the PBI, in which patients self-reported their pain severity. Relative *AVPRA* mRNA expression (2^−ddCt^ method) comparing IBS patients with low and high self-reported pain (defined as a PBI score of 0–2 [low pain, n = 13] or > 2 [high pain, n = 25], respectively).

The patients reporting high pain burden appeared to have higher *Avpr1a* expression than IBS patients reporting low pain burden or healthy controls, though this difference was not significant (Fig 9A). The relatively high variability may be due to the relatively small number of patients in the control group (n = 3) and difficulty recruiting this population (as screening colonoscopy is not indicated for pediatric patients). In a second study, *Avpr1a* expression was assessed in rectal biopsies from cohorts of adults with active (n = 6 with no pain, 9 with pain) or inactive (n = 6 with no pain, 7 with pain) UC, as well as healthy pain-free controls (n = 6) (Fig 9B). Relative mRNA expression (2^−ΔΔCt^ method) values were analyzed using 2 (Active vs Inactive) × 2 (Pain vs No Pain) ANOVA that revealed a significant main effect of pain status, but no other significant main effects or interactions (all *P* > .05). *Avpr1a* expression was significantly higher in biopsies from patients with pain than those without pain, regardless of their UC disease activity status (Fig 9B).

## Conclusions

The BL/6 inbred strain is ubiquitous in biomedical research serving as the primary reference or “normal” comparison strain for most mouse behavior and for the mouse genome assembly. However, the term BL/6 is often used to refer to a collection of highly genetically similar, but not identical, strains of mice,^48^ with little acknowledgment of the genotypic and phenotypic variations that exist between the closely related substrains. Inbred mouse strains are defined by the assumption of genome-wide homozygosity for all individual members and by the assumption of genomic fixation across time. Each BL/6 substrain satisfies these expectations, but multiple published studies comparing substrains have reported variability across various phenotypes, including motor coordination, behavior, and pain sensitivity.^49–53^ Only recently has the differential pain susceptibility across BL/6 strains been explored.^54–59^ Along these lines, we identified two BL/6 substrains with differential susceptibility to the development of VH and leveraged whole-genome sequencing^21^ to identify *Avpr1a* as the highest-priority candidate gene for differential VH susceptibility. *Avpr1a* belongs to a subfamily of G-protein-coupled receptors, and even though G-protein-coupled receptors have been found to modulate neuronal sensitization of the gut, the role of *Avpr1a* and its agonists has not previously been explored in basal colonic function or in VH susceptibility.

In line with prior work on the role of *Avpr1a* in somatic pain,^45^ the SNP differentiating BL/6J and BL/6NTac mice is in a proposed 5′ regulatory region outside of the *Avpr1a* coding sequence, pointing to a potential effect on gene expression rather than alterations in the encoded protein product. We confirmed a strain-dependent mRNA expression difference corresponding to VH susceptibility and a corresponding strain difference in functional sensitivity in response to the application of exogenous AVP, the *Avpr1a* agonist, via single-fiber recordings in an ex vivo colon-pelvic nerve single-fiber recording preparation. Moreover, *Avpr1a* expression was also higher in colorectal biopsies collected from UC patients with persistent abdominal pain when compared with disease-severity-matched UC patients with no pain, supporting the translational potential of *Avpr1a* genotype/expression as a risk factor for development of VH.

While *Avpr1a*/*Avpr1a* has been associated with somatic pain variability in prior work, the relationship between *Avpr1a* expression and somatic inflammatory pain was opposite to what we report for visceral pain as increased *Avpr1a* expression is associated with *hypo-*sensitivity for somatic pain, an effect that is sex- and stress-dependent in animals and human subjects.^44,45,60^ While these studies did not assess *Avpr1a* expression in all tissues comprising of the pain-processing pathway, from an area of inflammation to the brain, *Avpr1a* expression in the mouse DRG was inversely correlated with pain behavior (ie, lower *Avpr1a* expression in pain-sensitive mice). In contrast, the current data indicate that differential pain sensitivity between strains is not correlated with *Avpr1a* expression in DRG. It has also been previously shown that *Avpr1a* has some expression in small-diameter DRG neurons (referred to as putative nociceptors in the report) and that expression was increased in naïve mice with SNPs corresponding to higher expression of *Avpr1a* and lower pain intensity.^45^ Conversely, our data indicate that *Avpr1a* expression in enteric neurons of the colon is higher in VH-susceptible mice and that these neurons are more responsive to stimulation with the agonist AVP. The disagreement in the direction and location of prior findings and ours is likely due to the unique functional aspects of the distal colon compared with skin/distal somatic tissues. Relevant to our findings, *Avpr1a* may represent a novel point of convergence between VH following transient gastroenteritis and stress-induced VH that could explain the stress-induced pain exacerbations that many DGBI patients experience. Along these lines, endogenous vasopressin enters circulation downstream of the stress response and subsequent *Avpr1a* activation in the bowel results in vasoconstriction, alterations in water absorption in the colon, and abdominal pain/fecal urgency,^61–63^ mimicking the effects of exogenous vasopressin administration in the clinical setting (eg, to control GI bleeding, etc). For individuals with elevated colonic *Avpr1a/Avpr1a* expression and VH, vasopressin released in response to stress could result in significant exacerbation of VH through binding of AVP to *Avpr1a*, thereby linking this receptor to a potential mechanism for the clinically recognized stress-induced exacerbations of abdominal pain seen in IBS and other DGBIs.

Even though neuronal plasticity and broad alteration to the gut-brain axis have been implicated in the development of chronic abdominal pain, much of the prior work has focused on sensory neurons originating in pelvic, splanchnic, and ND ganglia due to the well-known relationship between innervating sensory neurons from the central nervous system (to the colon) and hypersensitivity.^12,64,65^ More recently, however, published data suggest a possible role for the ENS in pathological pain,^64,66,67^ though the mechanism of communication between the ENS and extrinsic neurons remains incompletely understood. While neither spinal nor ND ganglia sensory neurons were hyperresponsive to chemical stimulation by capsaicin and AVP in the absence of enteric neurons, enteric neurons were hyperresponsive to agonist exposure and the force-displacement response, which is under the influence of enteric neuron activity, was greater in the presence of AVP for ZYM-VH mice, we propose that communication between the ENS and extrinsic afferents may play a critical role in pain transmission under pathological conditions. Understanding a precise mechanism for enteric cell involvement in abdominal pain is beyond the scope of the present study. However, these data point to the potential utility of targeting *Avpr1a*/*Avpr1a* expression and/or function locally in the colon in the prevention and/or treatment of VH. In addition, *Avpr1a* genotype and/or expression may represent a potential biomarker for VH susceptibility with implications for the etiology of IBS and other DGBI conditions as well as for novel therapeutic interventions targeting this specific underlying mechanism.

The present study has certain limitations that should be noted. One notable constraint is that the experimental investigations were conducted solely using male mouse models, thereby limiting our ability to explore potential sex-specific differences. Consequently, caution must be exercised when extrapolating the study’s conclusions to female populations, as biological variations between male and female mice may exist and could impact the results. In addition, while there is no ongoing inflammation, the ZYM model may be considered a model of postinflammatory VH and whether this exact mechanism plays a role in individual differences in susceptibility to other forms of VH is yet to be determined. Furthermore, our immunobiological staining does not discriminate the location of *Avpr1a* in the distal colon between DRG endings and the enteric neurons, so our future work will include a higher-resolution exploration of *Avpr1a* location. Even with these caveats, the comprehensive studies reported herein propose a novel mechanism of genetic susceptibility to VH/persistent abdominal pain and point to a potential role for enteric neuron hyperresponsiveness in increased pain signal transmission from the colon in DGBIs.

## Supplementary Material

SupplementalFigures

## Figures and Tables

**Table 1.** Prioritized Candidate Genes Identified by Comparing BL/6NTac and BL/6J Substrains

Gene Symbol	Gene Name	Pain/Nociception Types That Have Been Associated in Mice	Number of Differential Snps/Consequence (So Term)	Snp(s) Identification
*Celsr1*	Cadherin, EGF LAG 7-pass G-type receptor 1	Neuropathic pain	2 SNPs/intron variants	rs51978880 rs236675812
*Clip3*	CAP-GLY domain containing linker protein 3	Thermal nociception	1 SNP/intron variant	rs3148686
*Aoah*	Acyloxyacyl hydrolase	Pelvic pain	1 SNP	Not identified on Ensembl Gene Browser
*Cacna2d1*	Calcium channel, voltage-dependent, alpha2/delta subunit 1	Mechanosensation	1 SNP	Not identified on Ensembl Gene Browser
*Avpr1a*	Arginine-vasopressin receptor 1A	Capsaicin and formalin inflammatory pain; acetic acid abdominal pain	1 SNP/noncoding transcript exon variant	rs29348001

NOTE. Only genes where a SNP exists within the 5′ flanking region, exon, introns, or 3′ UTR were included, which were defined as the gene and the surrounding DNA sequence up to 18K nucleotides outside the coding sequence on Ensembl Gene Browser. Note that only *Avpr1a has been associated with more than 1 type of pain*.

Abbreviation: CAP-GLY, Cytoskeleton-Associated Proteins- glycine rich; EGF, epidermal growth factor; LAG, laminin A G-type; UTR, untranslated region.

## Perspective:

This article presents evidence of Avpr1a as a novel candidate gene for VH in a mouse model of IBS. Avpr1a genotype and/or tissue-specific expression represents a potential biomarker for chronic abdominal pain susceptibility.
