# Supplementary material for: Identification of Arginine-Vasopressin Receptor 1a (Avpr1a/Avpr1a) as a Novel Candidate Gene for Chronic Visceral Pain Sheds Light on the Potential Role of Enteric Neurons in the Development of Visceral Hypersensitivity
Source: J Pain. Author manuscript; Available in PMC 2024 Nov 18. (PMC11571697; doi:10.1016/j.jpain.2024.104572)
Supplement: SupplementalFigures [file NIHMS2029461-supplement-SupplementalFigures.pdf]

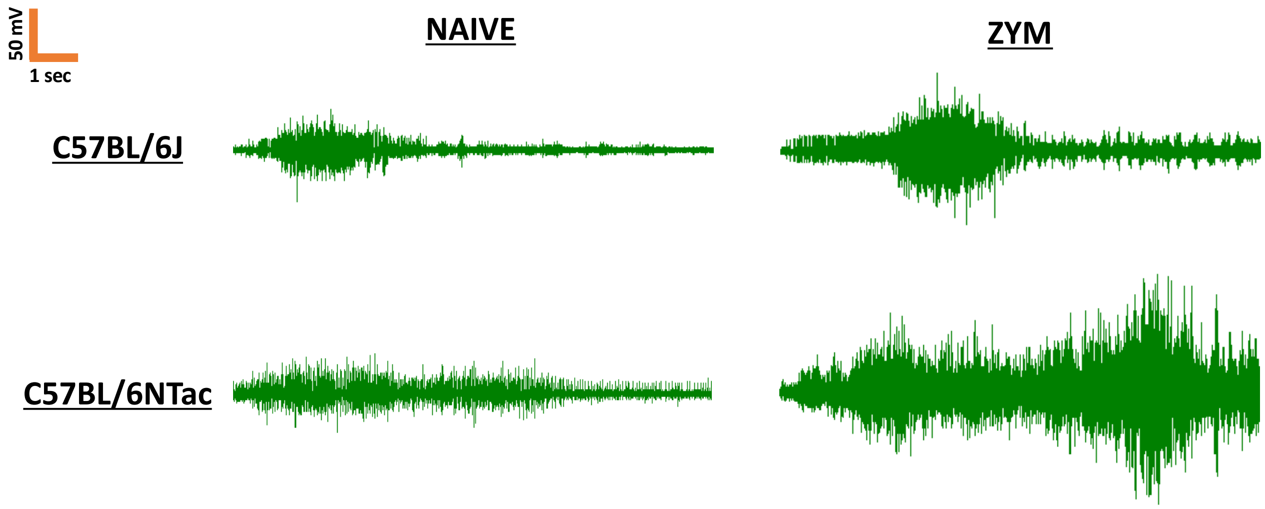

Supplemental Figure 1: Representative EMG recordings show a much greater increase in VMR response at 60 mmHg in C57BL/6NTac mice after ZYM treatment. VMR (EMG) to increasing colorectal balloon distension pressures (15 mmHg, 30 mmHg, 45 mmHg and 60 mmHg) in Naïve and ZYM treated BL/6NTac and BL/6J mice. At 60 mmHg, BL/6NTac mice after ZYM treatment exhibited increased EMG responses (VMR) during 10 second distension compared to BL/6J mice treated with intrarecal ZYM.

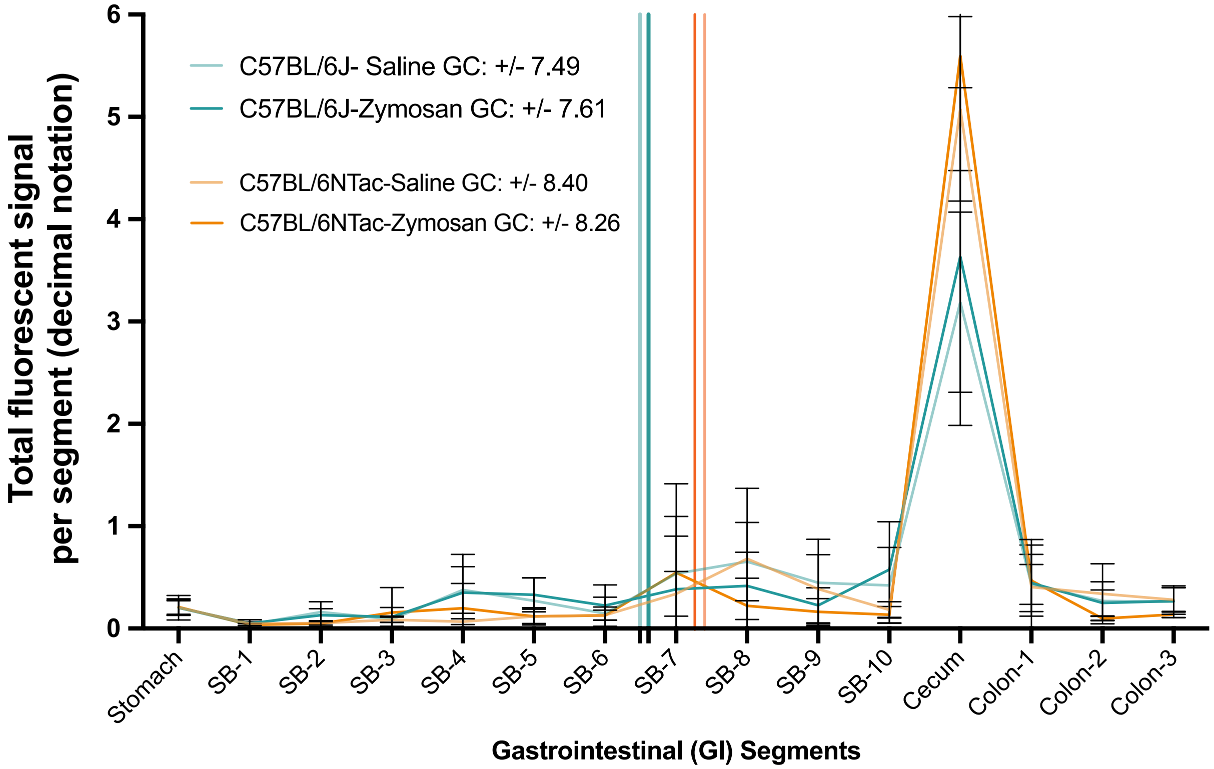

Supplemental Figure 2. ZYM treatment does not affect GIT in C57BL/6 substrains regardless of condition. A 2 X 2 ANOVA revealed no significant main effects or interactions. The geometric center (GC) of FITC-dextran distribution, the center of gravity for the distribution of the marker, is also not significant between either strain or treatment (indicated by the lines). No significance was found between main effects or interactions (all F's $\leq$ 3.051, all $p>0.05$) ($n = 10$).

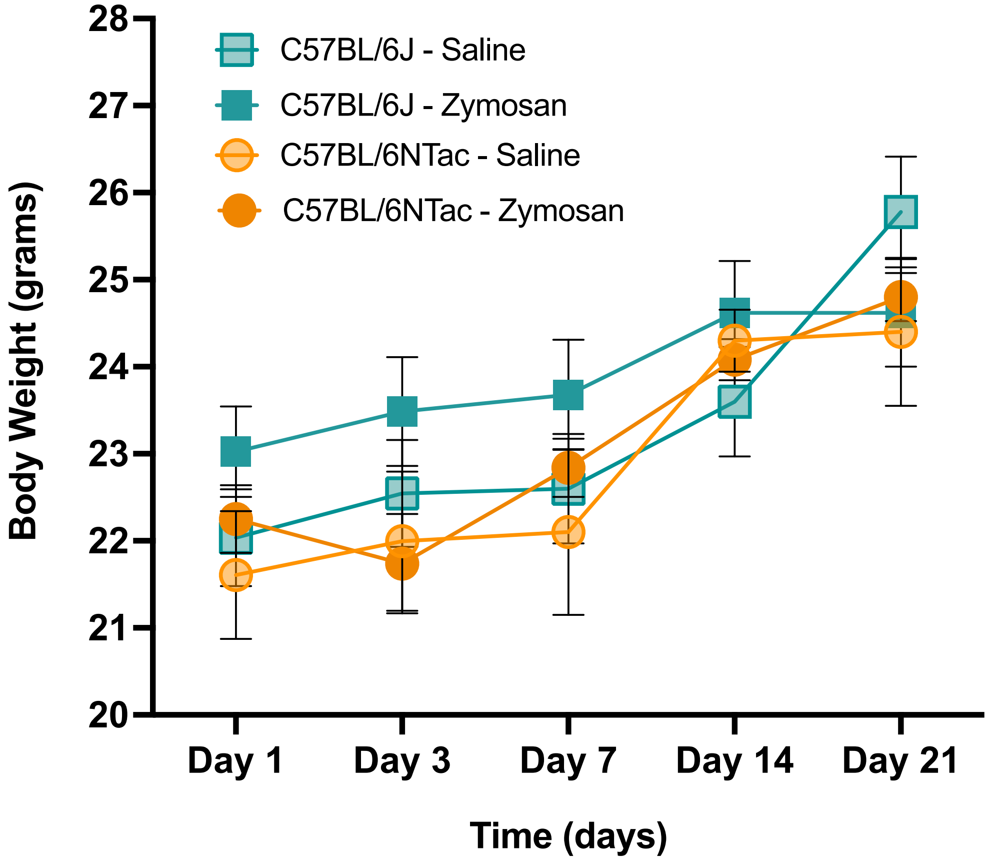

Supplemental Figure 3. ZYM does not affect body weight in BL/6 substrains. Body weight was measured during VH development (after ZYM/SAL treatment) for 21 days. *Teal = BL/6J, *Orange = BL/6NTac. 2 X 2 ANOVA with repeated measures revealed no significance in body weight. No other significance was found between main effects or interactions (all F's $\leq$ 1.173, all $p>0.05$) ($n = 5$).

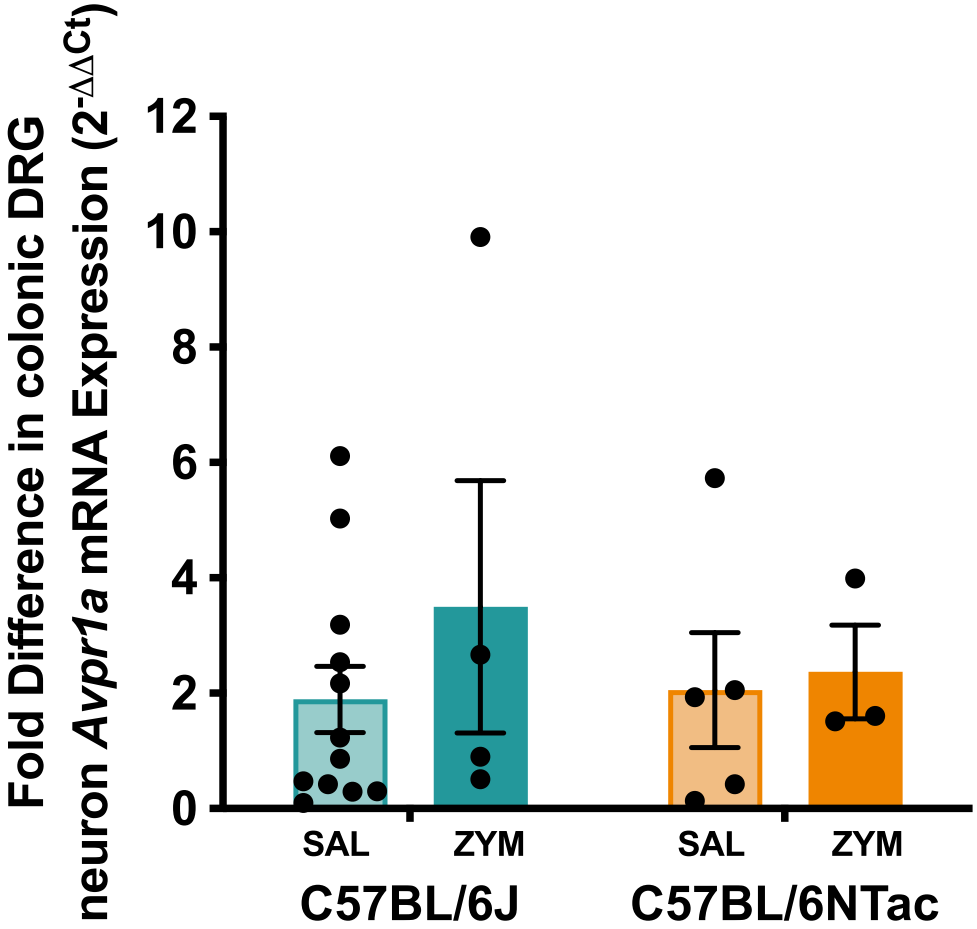

Supplemental Figure 4. Dorsal root ganglion (DRG) *Avpr1a* mRNA levels do not differ in SAL or ZYM-treated C57BL/6NTac and C57BL/6J mice. Relative mRNA expression of *Avpr1a* in pooled colon-specific thoracolumbar and lumbosacral DRG neurons did not differ by DRG-level, so data were combined. 2 X 2 ANOVA revealed no significant main effects or interactions on the expression of *Avpr1a* in colon-specific thoracolumbar and lumbosacral colon-specific primary sensory afferents (all F's $\leq$ 1.359, all $p>0.05$).

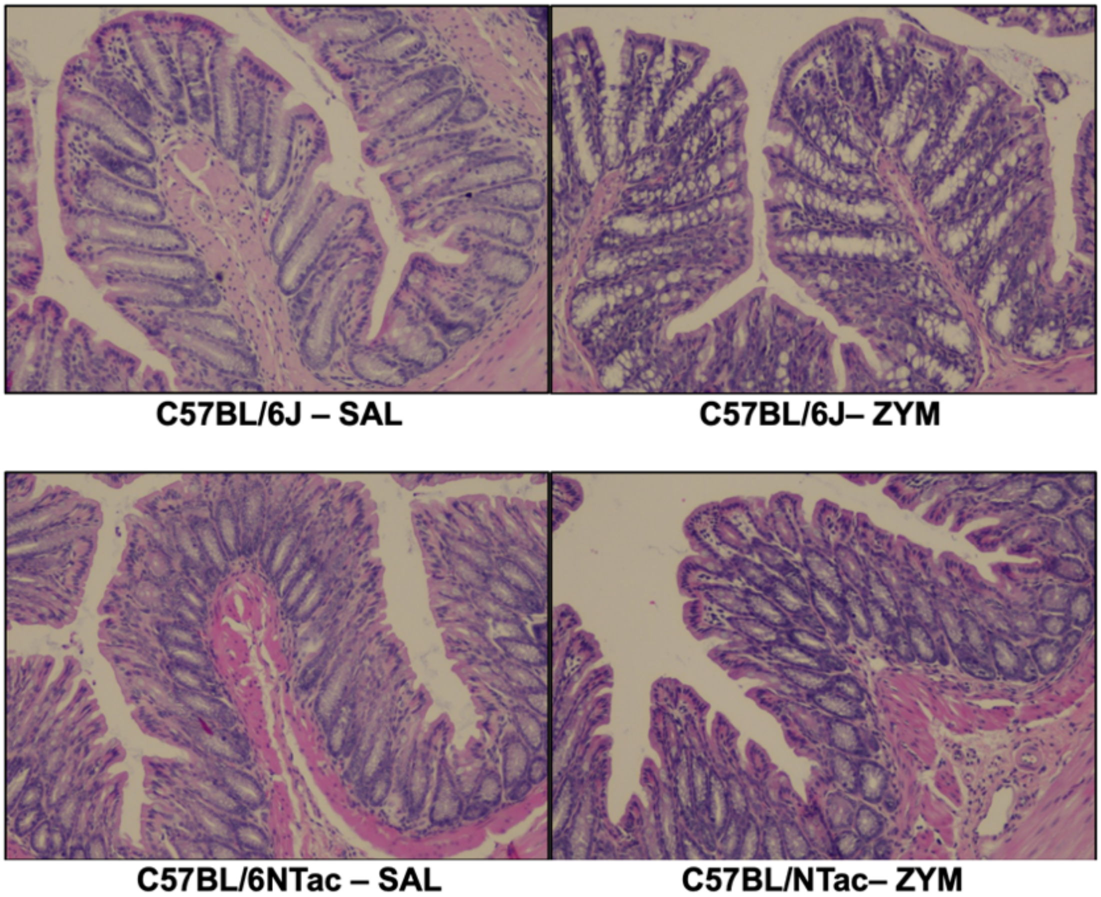

Supplemental Figure 5. **Distal colorectal morphology (inflammation) does not differ between C57BL/6J and C57BL/6NTac regardless of treatment.** Distal colon sections were collected at VH development. H&E staining showed no differences in morphology, including increased/expansion of existing lymphatic nodules or reduced integrity of epithelial crypts, between ZYM/SAL treated BL/6J and BL/6NTac mice (n = 5).

A) B)

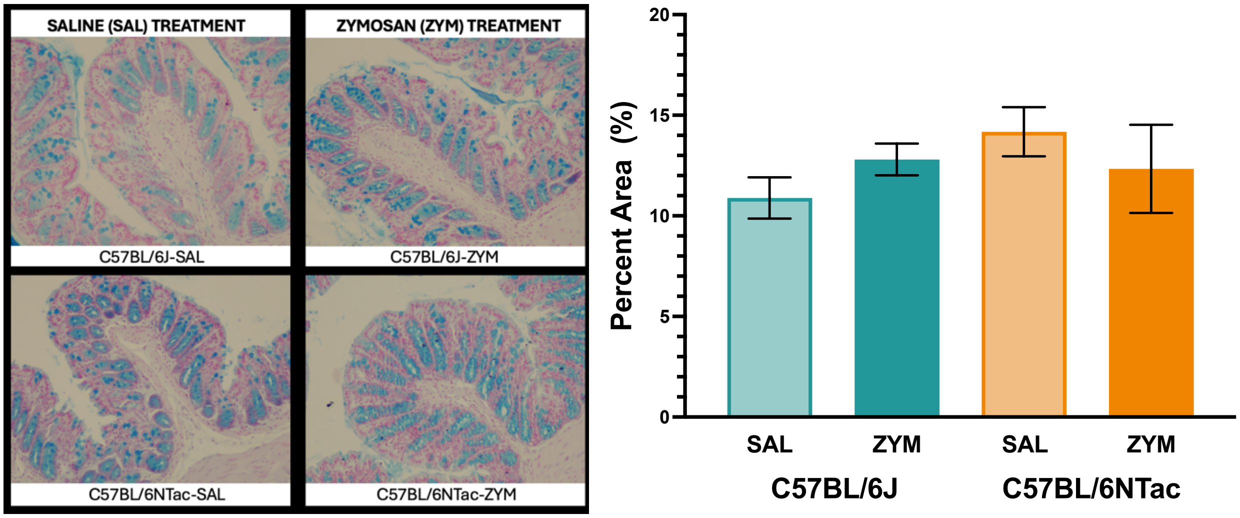

Supplemental Figure 6. Distal colorectal morphology (Goblet cell and mucus production) does not differ between C57BL/6J and C57BL/6NTac mice regardless of ZYM treatment. (A) Representative Alcian blue stains for each strain (BL/6NTac vs. BL/6J) and condition (ZYM vs. SAL). **(B)** Percent area of mucin cells was calculated using ImageJ/Fiji). 2 X 2 ANOVA showed no significance in percent area of mucin cells between either strain or condition (all F's $\leq$ 17.653 , all $p>0.05$) ($n = 5$).

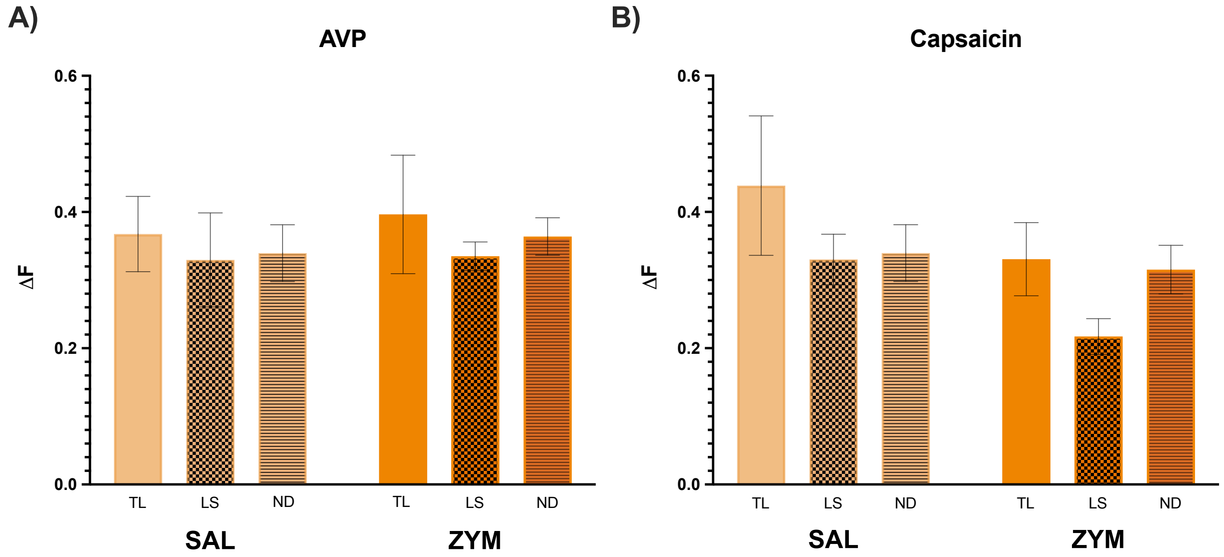

Supplemental Figure 7. In vitro Ca^{2+} imaging of extrinsic colon neurons in C57BL/6NTac mice (SAL vs ZYM) revealed no significant difference in Ca^{2+} response ($\Delta F_{340/380}$) in response to stimulation with AVP or Capsaicin. In vitro Ca^{2+} imaging of retrogradely labeled extrinsic neurons (thoracolumar DRGs = TL; lumbosacral DRGs = LS; nodose (vagus) ganglia = ND) revealed no significant differences in Ca^{2+} influx (ΔF) in response to AVP and CAP compared to SAL controls . (all $p>0.05$) (n=5).

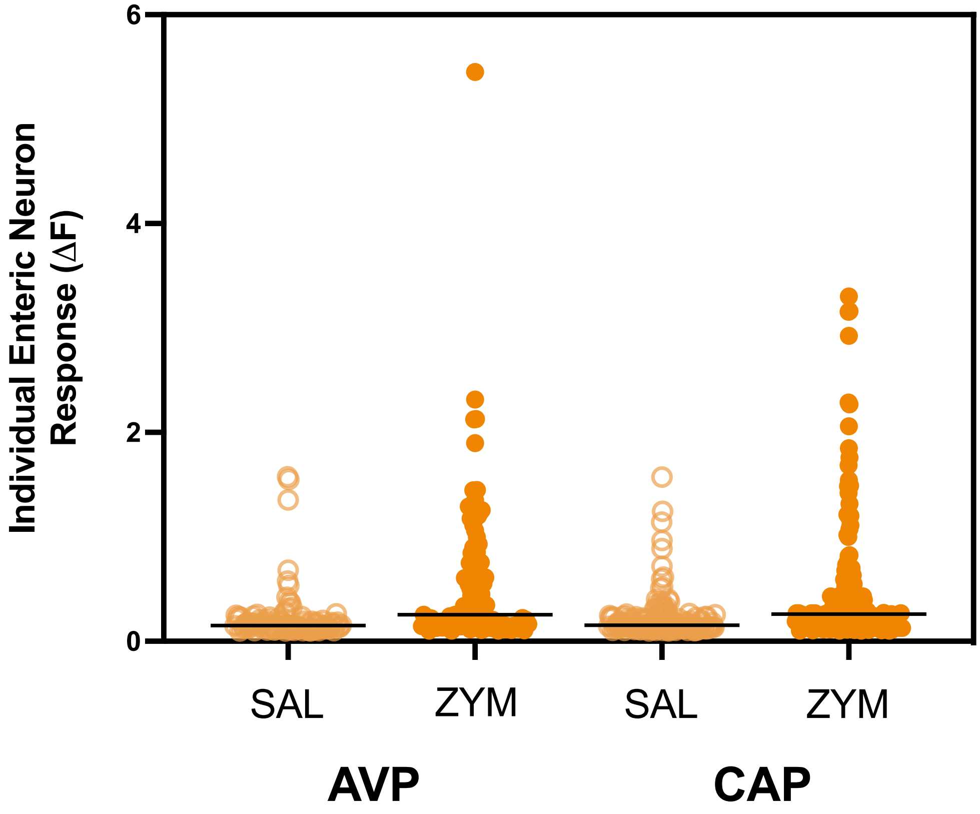

Supplemental Figure 8. Individual Ca^{2+} transient responses of flourescent response/condition (ΔF) in response to AVP and Capsaicin (CAP) in enteric neurons from C57BL/6NTac mice (ZYM vs. SAL). Visual representation of individual enteric neuron Ca^{2+} transient responses from **Figure 8**. Individual data points show heterogenatity of enteric neuron Ca^{+2} reponse between treatment condition and agonist. Lines represent the mean ΔF. Refer to Figure 8 for statisical analysis. n = 5 mice/condition.

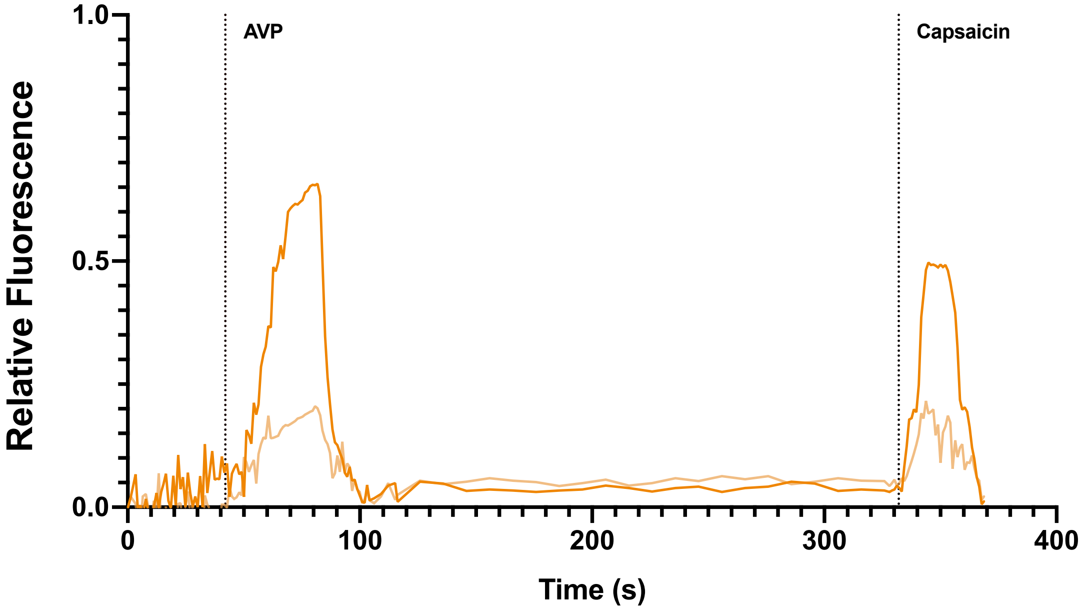

Supplemental Figure 9. Representitive Ca^{2+} imaging traces from enteric neurons. Traces of enteric neurons from BL/6NTac-ZYM (dark orange line mice vs BL/6NTac-SAL (light orange line) mice show differences in response of AVP and CAP over the cycle of agonist exposure. Dotted lines indicate the timing of agonist application to individual neurons (n = 5).
